# Hedgehog signaling activation induces stem cell proliferation and hormone release in the adult pituitary gland

**DOI:** 10.1038/srep24928

**Published:** 2016-04-25

**Authors:** Joanna Pyczek, Rolf Buslei, David Schult, Annett Hölsken, Michael Buchfelder, Ina Heß, Heidi Hahn, Anja Uhmann

**Affiliations:** 1 Institute of Human Genetics, Tumor Genetics Group, University of Göttingen, Germany; 2 Institute of Neuropathology, Friedrich-Alexander University Erlangen-Nürnberg (FAU), Germany; 3 Department of Neurosurgery, Friedrich-Alexander University Erlangen-Nürnberg (FAU), Germany

## Abstract

Hedgehog (HH) signaling is known to be essential during the embryonal development of the pituitary gland but the knowledge about its role in the adult pituitary and in associated tumors is sparse. In this report we investigated the effect of excess Hh signaling activation in murine pituitary explants and analyzed the HH signaling status of human adenopituitary lobes and a large cohort of pituitary adenomas. Our data show that excess Hh signaling led to increased proliferation of Sox2^+^ and Sox9^+^ adult pituitary stem cells and to elevated expression levels of adrenocorticotropic hormone (Acth), growth hormone (Gh) and prolactin (Prl) in the adult gland. Inhibition of the pathway by cyclopamine reversed these effects indicating that active Hh signaling positively regulates proliferative processes of adult pituitary stem cells and hormone production in the anterior pituitary. Since hormone producing cells of the adenohypophysis as well as ACTH-, GH- and PRL-immunopositive adenomas express SHH and its target *GLI1*, we furthermore propose that excess HH signaling is involved in the development/maintenance of hormone-producing pituitary adenomas. These findings advance the understanding of physiological hormone regulation and may open new treatment options for pituitary tumors.

The Hedgehog (HH) signaling pathway is closely linked to developmental processes, organ patterning, tissue and stem cell maintenance, cell differentiation processes, cell proliferation, regenerative responses after injury and cancer formation^1^,^2^,^3^. Binding of the ligand HH to its receptor Patched 1 (PTCH) results in activation of the pathway by suspension of the PTCH-dependent inhibition of Smoothened (SMO). Subsequently the GLI transcription factors (GLI2, GLI3) transfer the activation signal into the nucleus and regulate the expression of several GLI target genes. In a positive and negative feedback loop these targets encompass *GLI1* and *PTCH*, respectively. Abnormal decrease of HH signaling results in developmental defects whereas an increase results in cancer formation (reviewed in^1^).

HH signaling is also essential for the development of the pituitary. For example, holoprosencephaly patients with inactivating mutations in Sonic HH (SHH) suffer from an agenesis of this organ^4^. Similar observations have been made in Shh knockout mice^5^,^6^. Additionally, inactivating *GLI2* mutations have been associated with hypopituitarism and pituitary malformations^7^,^8^. In contrast excess Hh signaling activity due to overexpression of Shh results in pituitary hyperplasia in mice^9^. Moreover, inactivating *PTCH* mutations may affect the hormone homeostasis of the pituitary since patients with heterozygous *PTCH* germline mutation (Gorlin-Goltz-Syndrome) as well as heterozygous *Ptch* knockout mice occasionally develop acromegaly-like symptoms^10^,^11^,^12^,^13^,^14^,^15^,^16^,^17^,^18^,^19^.

Besides the involvement of HH signaling pathway in pituitary development, several links indicate that this pathway is also involved in the maintenance and hormone homeostasis of this organ. Thus, the human anterior pituitary expresses SHH and GLI1, which suggest that HH signaling plays a role in hormone secretion^20^,^21^.

Hormone producing cells of the frontal pituitary lobe are the origin of the vast majority of pituitary adenomas (PA). These tumors constitute about 10 to 15% of all intracranial neoplasms and in general represent benign epithelial lesions^22^. Recently it has been suggested that SHH maintain pituitary tumor cells in a non-proliferative state. Consequently, HH pathway activity was proposed to prevent the development of PA^21^. However, the proof of this hypothesis is missing.

We here assessed the effect of activation and inactivation of Hh signaling on morphology, hormone expression/release and proliferation of pituitary explants and single cells isolated from *Ptch*^*flox/flox*^ *CreERT2* mice^23^. In addition, we analyzed the activation status of HH pathway in the human adenopituitary (qRT-PCR: n = 12, specific immunohistological stainings: n = 15) and in a large cohort of human pituitary tumors (qRT-PCR: n = 48, specific immunohistological stainings: n = 96). In contrast to former reports, our results demonstrate that active Hh signaling induces proliferation of Sox2^+^ and Sox9^+^ adult pituitary stem cells and hormone release in the adult pituitary gland. Finally our data suggest that activation of the HH pathway may be involved in the formation and/or maintenance of pituitary tumors. Therefore, inhibition of Hh signaling could be a promising new target for the treatment of aggressive PA.

## Results

### Hh signaling activation induces hormone secretion and proliferation of Sox2^+^ and Sox9^+^ adult pituitary stem cells in murine pituitaries

**Figure 1:**
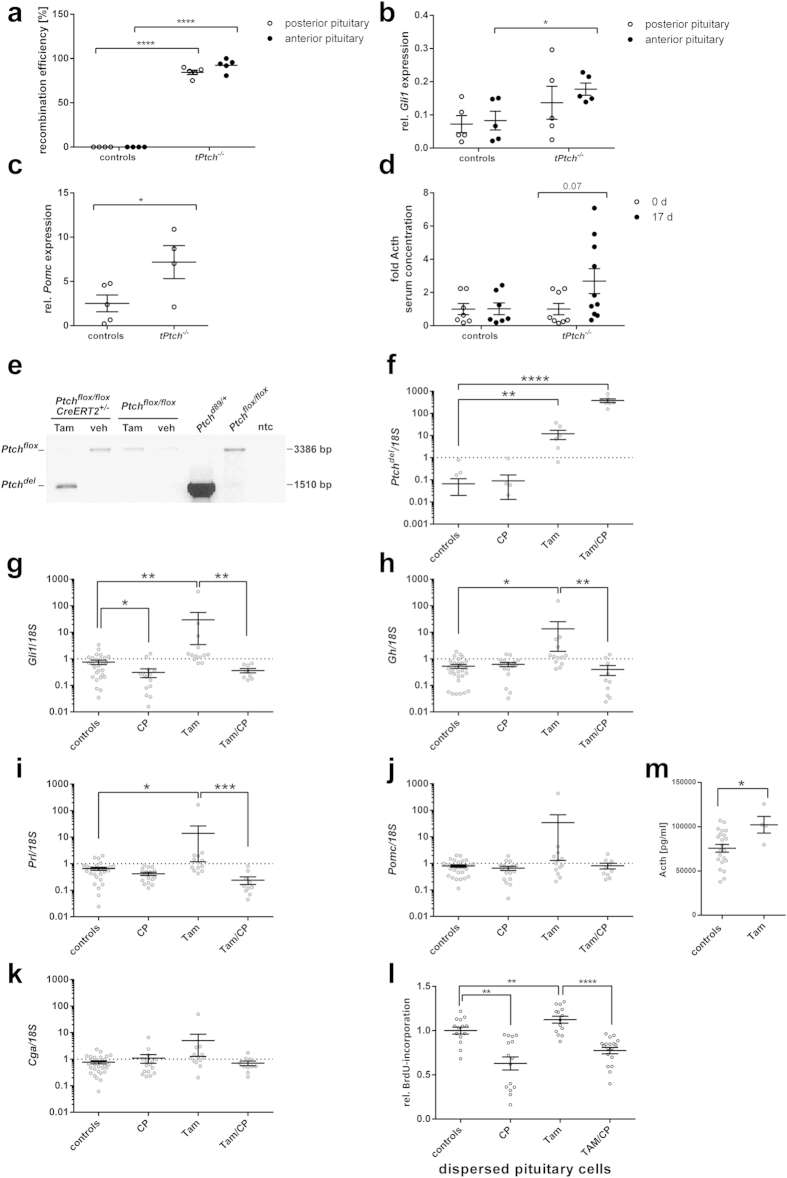
*Ptch* depletion leads to activation of Hh signaling and to increased expression/release of pituitary hormones and proliferation. (**a**–**d**) *In vivo* analyses: (**a**) Recombination efficiencies at the genomic *Ptch*^*flox*^ locus, (**b**) *Gli1* expression levels in posterior (open circles) and anterior pituitary glands (black circles) and (**c**) *Pomc* expression levels in anterior pituitary glands of *tPtch*^−/−^ (2 females, 2 males) and control mice (2 females, 3 males) 17 days after the first tamoxifen-injection. (**d**) Fold change of Acth blood serum concentration of *tPtch*^−/−^ mice before (4 females, 4 males; open circles) and 17 days after the first tamoxifen-injection (5 females, 5 males; black circles) in comparison to control mice (3 females, 4 males; tamoxifen-treated *Ptch*^*flox/flox*^ and vehicle-treated *Ptch*^*flox/flox*^*CreERT2*^+/−^ mice). (**e**–**m**) *ex vivo* analyses: (**e**) PCR-based recombination analysis of the genomic *Ptch*^*flox*^ locus, (**f**–**k**) relative *Ptch*^*del*^, *Gli1*, *Gh*, *Prl*, *Pomc* and *Cga* expression levels and (**l**) BrdU incorporation assays of tamoxifen-treated *Ptch*^*flox/flox*^*CreERT2*^+/−^ (Tam) and control (**e**–**k**) pituitary explants or (**l**) dispersed cells with or without cyclopamine treatment (CP). (**e**) Amplification of the *Ptch*^*flox*^ locus results in a 3386 bp fragment. Recombination of the *Ptch*^*flox*^ locus leads to amplification of a 1510 bp fragment due to the deletion of *Ptch* exons 8 and 9^23^. ntc, no template control. (**f**–**k**) n_controls_ = 28 (14 females, 14 males), n_CP_ = 16 (7 females, 9 males), n_Tam_ = 13 (7 females, 6 males), n_Tam/CP_ = 10 (5 females, 5 males). Data shown in (**l**) represent 6 independent experiments. (**f**–**l**) Controls include vehicle-treated *Ptch*^*flox/flox*^ *CreERT2*^+/−^, tamoxifen- or vehicle-treated *Ptch*^*flox/flox*^ and untreated pituitary glands/cells of both genotypes. (**m**) Quantification of the Acth concentration in supernatants of *ex vivo* recombined *Ptch*^*flox/flox*^*CreERT2*^+/−^ (Tam) and control pituitaries. Circles indicate Acth concentrations of supernatant conditioned from 3 pituitaries of the same genotype. Controls include vehicle-treated *Ptch*^*flox/flox*^*CreERT2*^+/−^ and tamoxifen- or vehicle-treated *Ptch*^*flox/flox*^ pituitary glands. Acth serum levels in (**d**), *Gli1*, *Gh*, *Prl*, *Pomc* and *Cga* expression levels and BrdU incorporation of the controls were set to 1. (**a**–**d**,**f**–**l**) Circles indicate biological replicates measured in triplicates. Horizontal lines, mean^+/−^ standard error of the mean (SEM); *p < 0.05, **p < 0.01, ***p < 0.001, ****p < 0.0001.

To investigate if activation of Hh signaling triggers expression of pituitary hormones, we analyzed pituitaries from *Ptch*^*flox/flox*^*CreERT2*^+/−^ mice 17 d after tamoxifen-mediated induction of a biallelic *Ptch* deletion (*tPtch*^−/−^ mice)^23^. The floxed *Ptch*^*flox*^ loci of the posterior and the anterior *tPtch*^−/−^ pituitaries were efficiently deleted (Fig. 1a), which resulted in significantly elevated *Gli1* transcription (Fig. 1b, p = 0.023) and thus in activation of Hh signaling in the respective glands. Additionally, the *Pomc* expression levels of the pituitaries and the Acth serum levels of *tPtch*^−/−^ mice were increased compared to the controls (Fig. 1c,d).

**Figure 2:**
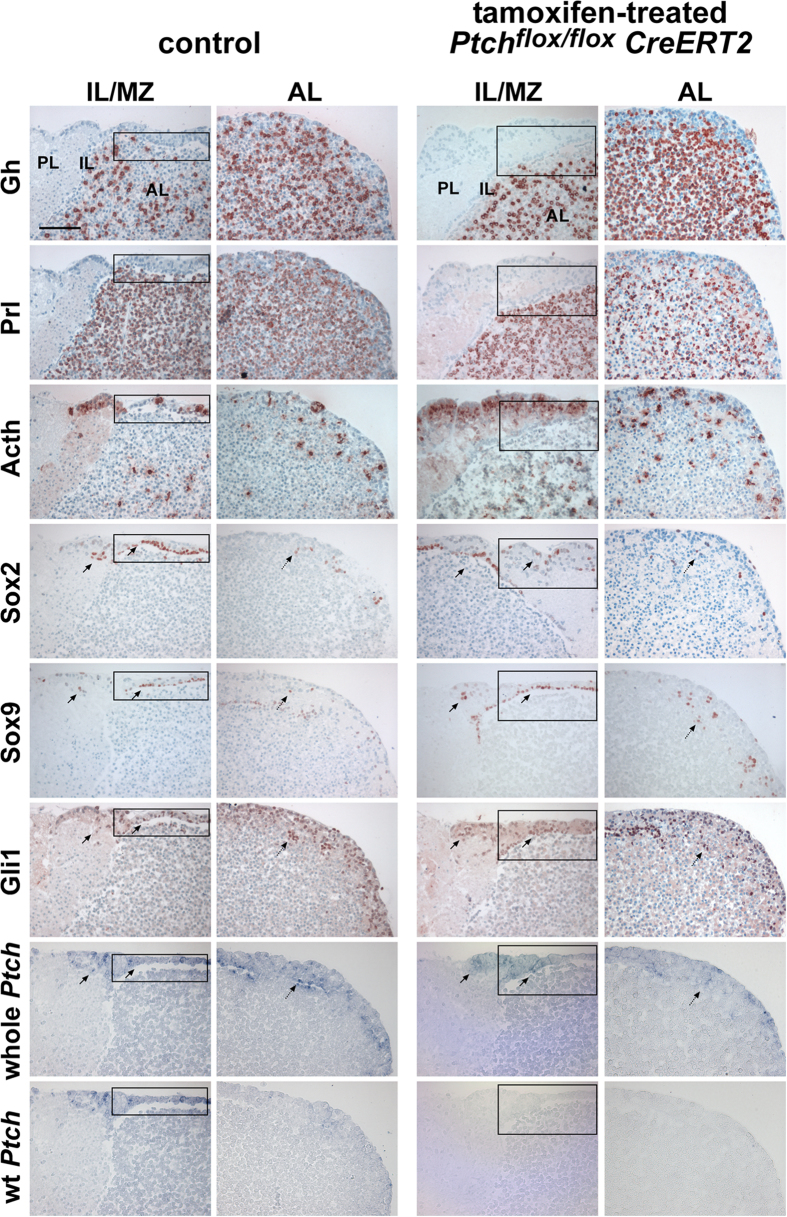
Distribution pattern of Gh, Prl, Acth, Sox2, Sox9, Gli1 and *Ptch* in *ex vivo* cultured *Ptch*^*flox/flox*^ *CreERT2*^+/−^ and control pituitary glands. Immunohistochemical analyses of Gh, Prl, Acth, Sox2, Sox9 and Gli1 and *in situ* hybridization for detection of *Ptch* transcripts on serial sections of control and *Ptch*-mutant (tamoxifen-treated *Ptch*^*flox/flox*^ *CreERT2*^+/−^) *ex vivo* cultured murine pituitary explants. Arrows indicate overlapping Sox2, Sox9, Gli1 and *Ptch* expression pattern in the intermediate lobe (IL) and the marginal zone (squares). Dotted arrows indicate overlapping Sox2, Sox9, Gli1 and *Ptch* expression pattern in the anterior lobe (AL). For detection of whole *Ptch* transcripts a 477 bp riboprobe was used, which identifies mutant and wt *Ptch* transcripts simultaneously. A second 250 bp riboprobe detects exclusively wt *Ptch* transcripts^48^. Tamoxifen-treated *Ptch*^*flox/flox*^ *CreERT2*^+/−^ glands express only mutant *Ptch* transcripts, as signals were exclusively obtained using the *Ptch* 477 bp riboprobe. PL, posterior lobe; Acth, adrenocorticotrophic hormone; Gh, growth hormone; Prl, prolactin. Scale bar: 100 μm.

Since the animals are in a very poor general condition 17 d after induction of the *Ptch* deletion^23^, the elevated Acth levels in *tPtch*^−/−^ mice could be stress-induced or correlated with activation of the Hh signaling pathway. To prevent potential hormonal feed-back loops that may have distorted the results concerning the direct influence of Hh signaling on hormone production, we isolated *Ptch*^*flox/flox*^*CreERT2*^+/−^ pituitaries and induced the *Ptch* mutation *ex vivo* by administrating tamoxifen (for verification of successful culture see Fig. 2 showing immunohistological stainings of cultured explants). This resulted in recombination of the *Ptch*^*flox*^ loci (*Ptch*^*del*^) (Fig. 1e), a significant increase of mutant *Ptch* transcripts (*Ptch*^*del*^) (Fig. 1f) (p = 0.0013) and a loss of wt *Ptch* expression in *Ptch*^*flox/flox*^*CreERT2*^+/−^ compared to control pituitaries (Fig. 2). As expected, the mutation furthermore resulted in elevated *Gli1* expression levels and thus activation of Hh signaling in *Ptch*^*flox/flox*^*CreERT2*^+/−^ glands (Fig. 1g). Similar results were obtained after incubation of normal murine pituitaries with rShh-N, which likewise resulted in an upregulation of *Gli1* and wt *Ptch* transcription, however to a much lesser extent (Suppl. Fig. S1a,b). When the glands were treated with cyclopamine, the *Gli1* expression levels were considerably reduced in tamoxifen-treated *Ptch*^*flox/flox*^*CreERT2*^+/−^, in rShh-N-treated and solvent-treated organs (Fig. 1g; Suppl. Fig. S1b). Most interestingly, *Ptch* deletion also resulted in a tendency towards increased *Pomc* and *Glycoprotein hormones alpha chain* (*Cga*) transcript levels (Fig. 1j,k), in a significantly elevated Acth release (Fig. 1m) (p = 0.023) and upregulation of *Gh* and *Prl* expression (Fig. 1h,i). These effects were abrogated by cyclopamine treatment (Fig. 1h–k), indicating a direct involvement of Hh signaling in the transcriptional activation of these genes. In contrast, the expression levels of *Oxt*, *Tsh*β, *Lh*β and *Fsh*β were not altered by *Ptch* depletion, cyclopamine or rShh-N treatment (Suppl. Fig. S2).

**Figure 3:**
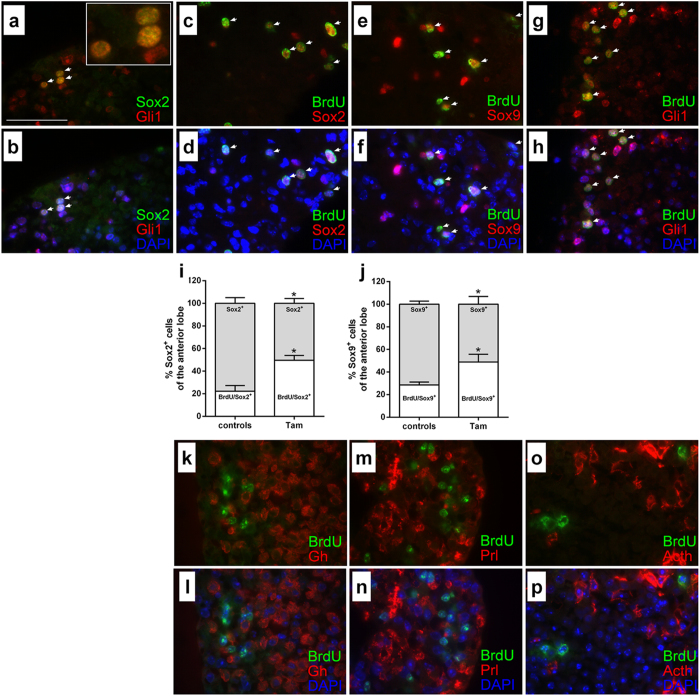
*Ex vivo Ptch* depletion in murine pituitaries results in proliferation of Sox2^+^/Gli1^+^ and Sox9^+^ adult pituitary stem cells. (**a**,**b**) Representative picture of a wt murine pituitary explant stained against Sox2 and Gli1. (**a**) Overlay of Sox2 (green color) and Gli1 (red color). (**c**–**h** and **k**–**p**) Representative pictures of wt murine pituitary explants stained against BrdU and (**c**,**d**) Sox2, (**e**,**f**) Sox9, (**g**,**h**) Gli1, (**k**,**l**) Gh, (**m**,**n**) Prl, (**o**,**p**) or Acth. (**c**,**e**,**g**,**k**,**m**,**o**) Overlays of BrdU (green color) and staining against the indicated secondary antigen (red color). (**b**,**d**,**f**,**h**,**l**,**n**,**p**) Same pictures as in (**a**,**c**,**e**,**g**,**k**,**m**,**o**) including the DAPI-channel (blue color). White arrows in (**a**–**h**) indicate Sox2^+^/Gli1^+^ cells or BrdU^+^ proliferating Sox2^+^, Sox9^+^ and Gli1^+^ cells respectively. (**i**,**j**) Relative quantification of proliferating (white bars, BrdU/Sox2^+^ or BrdU/Sox9^+^) and non-proliferating (grey bars, Sox2^+^ or Sox9^+^) (**i**) Sox2^+^ and (**j**) Sox9^+^ cells in the anterior pituitary of double immunofluorescent stained tamoxifen-treated *Ptch*^*flox/flox*^*CreERT2*^+/−^ (Tam) and control pituitary gland explants. Analysis is based on the absolute cell numbers given in Suppl. Fig. S3. (**i**) n_controls_ = 4 (1 female, 3 males), n_Tam_ = 7 (6 females, 1 male), (**j**) n_controls_ = 4 (1 female, 3 males), n_Tam_ = 6 (5 females, 1 male). Mean+/−SEM; *p < 0.05. Scale bar: 50 μm.

Immunohistological examinations of short-term cultured explants of tamoxifen-treated *Ptch*^*flox/flox*^*CreERT2*^+/−^ and control pituitaries revealed no obviously changed expression patterns of Gh, Prl and Acth, the adult pituitary stem cell marker Sox2 and Sox9^24^, Gli1, and *Ptch* (Fig. 2). Remarkably, the expression of Sox2, Sox9, Gli1 and *Ptch* overlapped in cells of the intermediate zone, the marginal zone and the anterior lobe although Gli1 or *Ptch* positivity was not exclusively restricted to Sox2^+^ or Sox9^+^ cells (Fig. 2). Beyond that double immunofluorescent stainings against Sox2 and Gli1 revealed that all Sox2^+^ cells of the intermediate lobe, the marginal zone (data not shown) and the anterior lobe also are Gli1^+^ (Fig. 3a,b). However Gli1 expression was not exclusively restricted to Sox2^+^ cells (Fig. 3a,b).

5′-bromo-2′-deoxyuridine (BrdU) incorporation assays of single pituitary cell cultures revealed a considerably higher proliferation rate of tamoxifen-treated *Ptch*^*flox/flox*^*CreERT2*^+/−^ but not of rShh-N-treated cells compared to the controls (Fig. 1l, Suppl. Fig. S1g). Moreover, cyclopamine inhibited the proliferation of tamoxifen-treated *Ptch*^*flox/flox*^*CreERT2*^+/−^ and control cells (Fig. 1l, Suppl. Fig. S1g).

To further characterize the proliferating cell population, tamoxifen-treated *Ptch*^*flox/flox*^*CreERT2*^+/−^ explants were cultured in BrdU-containing medium and double immunoflourescent stainings against BrdU in combination with anti-Sox2, anti-Sox9, anti-Gli1, anti-Gh, anti-Prl anti-Acth and anti-Pomc antibodies were conducted. This approach revealed that all BrdU^+^ cells of the intermediate lobe, the marginal zone (data not shown) and the anterior lobe showed a distinct co-expression of Sox2, Sox9 or Gli1 (Fig. 3c–h). In contrast, endocrine cells (e.g. Gh^+^, Prl^+^, Acth^+^ and Pomc^+^) never showed BrdU positivity (Fig. 3k–p for BrdU/Gh, BrdU/Prl, BrdU/Acth staining). Quantification of the proliferating cells revealed more BrdU^+^ Sox2^+^ or BrdU^+^ Sox9^+^ cells in the anterior lobe of tamoxifen-treated *Ptch*^*flox*/*flox*^*CreERT2*^+/−^ explants (Fig. 3i,j, Suppl. Fig. S3c,f), although the absolute numbers of Sox2^+^ and Sox9^+^ proliferating cells did not change (Suppl. Fig. 3a,d). Due to the fact that our experimental setup is sufficient to induce a statistically significant increase in the numbers of pituitary stem cells, but not of differentiated progeny^25^ these data exclude that enhanced Acth, Gh and Prl expression levels of *Ptch*-depleted pituitary glands result from elevated numbers of endocrine cells. It furthermore stresses our conclusion that Hedgehog signaling not only induces proliferation of Sox2^+^ cell but also activates the expression of Acth, Gh and Prl in already existing differentiated endocrine cells.

### Gli-dependent activation of the murine and human Pomc/POMC promoter

Gli1 and Sox2 expression patterns largely overlap in the intermediate lobe, the marginal zone and the anterior lobe, but Gli1 positivity was not restricted to Sox2^+^ cells (see Fig. 3a,b). This suggests that Hh signaling might be also implicated in the function of endocrine cells. Sequence analyses of the mouse, rat and human *Pomc/POMC* promoter revealed two Gli binding sites upstream of the first ATG (Suppl. Fig. S4a). To validate our finding from the *ex vivo* explant experiments we tested whether activation of Hh signaling directly induces Acth-secretion in the murine PA cell line AtT-20 that has been widely used as a model system for Acth-expressing pituitary cells^20^,^21^,^26^,^27^,^28^. However, AtT-20 cells express only low levels of *Ptch* and no *Gli1* or *Gli2*, and an induction of these genes upon Shh stimulation was not possible (data not shown). Nevertheless Gli1 or Gli2 overexpression resulted in transcriptional activation of the *Ptch* gene (Suppl. Fig. S4b), higher *Pomc* transcription and activation of the human *POMC* promoter (Suppl. Fig. S4c,e). Moreover Gli1 or Gli2 overexpression – contrary to Shh treatment – did not alter the proliferative capacity of AtT-20 cells (Suppl. Fig. S4d) which is in line with the observation that *Ptch* depletion does not induce proliferation of Acth-expressing cells in pituitary explants (see Fig. 3o,p). Besides, these results indicate that Shh induces proliferation in AtT-20 cells in a Gli1/Gli2-independent manner.

### Expression of SHH and GLI1 in corticotropic, somatotropic and lactotropic cells of the human adenohypophysis and related adenoma subtypes

**Figure 4:**
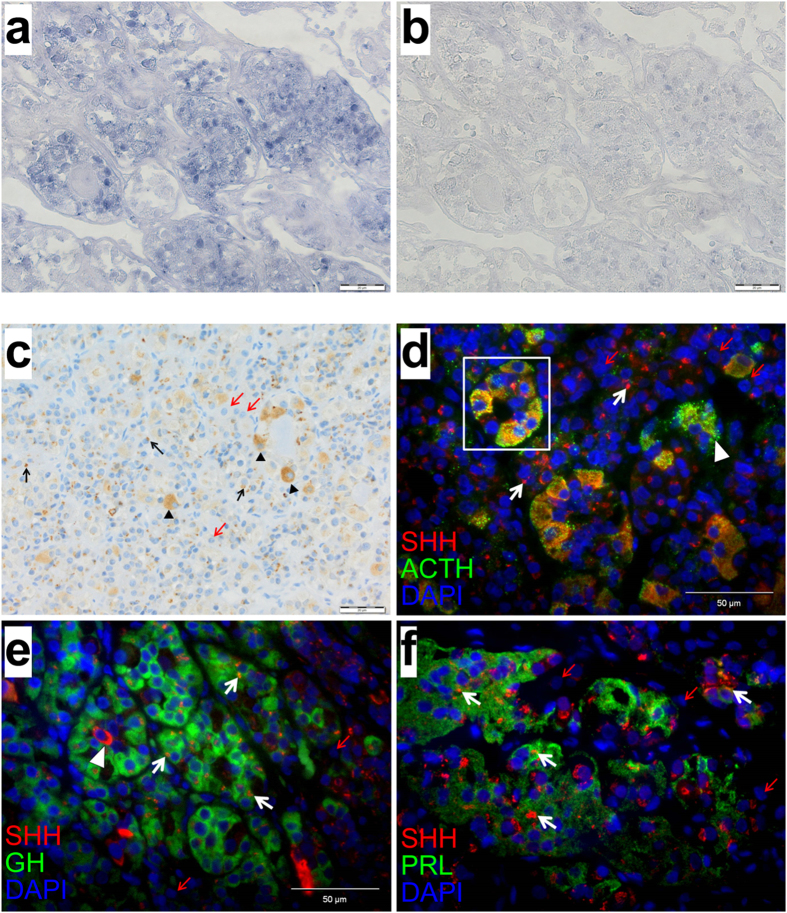
*GLI1* expression and SHH protein localization in the human pituitary gland. (**a**,**b**) Representative *GLI1 in situ* hybridization, (**c**) SHH immunohistochemical staining and (**d–f**) double immunofluorescent stainings against SHH and (**d**) ACTH, (**e**) GH or (**f**) PRL in the human adenohypophysis. (**a**) *GLI1* is expressed in several endocrine cells of the human adenohypophysis indicating activation of the HH signaling pathway. Cells are organized in small lobules, being characteristic for the physiological anatomy. (**b**) Serial section hybridized with *GLI1* sense riboprobes served as negative control. (**c–f**) Particular cells of the human adenohypophysis either show a distinct, homogenous and particularly granular SHH staining pattern (arrowheads), or a circumscribed and dot-like pattern (black arrows in **a** and white arrows in (**d–f**). SHH negative cells are exemplarily marked with red arrows. (**d–f**) The majority of ACTH expressing cells is SHH positive (yellow color, square, granular staining pattern). Several GH or and PRL positive cells (green color) show a distinct perinuclear, dot-like localization of SHH (red color, white arrows). ACTH, adreno-corticotrophic hormone; GH, growth hormone; PRL, prolactin. Scale bar in (**a–c**) 20 μm; Scale bar in (**d–f**) 50 μm.

To transfer our results to the human situation we analyzed the HH signaling activity in 15 normal adenopituitaries by *GLI1 in situ* hybridization (Fig. 4a,b) and/or anti-SHH immunostaining using an antibody generated against the N-terminal part of SHH protein (Fig. 4c–f). Cells throughout the adenohypophysis homogenously expressed *GLI1* (Fig. 4a,b) and stained positive for SHH to, however, variable extent. We found cell populations displaying an intense cytoplasmic and predominantly granular SHH staining pattern as well as cells with restricted immunoreactivity to particularly perinuclear structures (Fig. 4c). Double immunofluorescent stainings using antibodies against SHH and several hormones produced in the adenohypophysis (ACTH, GH and PRL) support former studies^21^ that ACTH-expressing (corticotropic) cells show a distinct, homogenous SHH immunoreactivity (Fig. 4d). However, besides co-localization of SHH and corticotrophs circumscribed, perinuclear, dot-like SHH positivity in obviously lactrotrophs and somatotrophs was observed (Fig. 4e,f). Since binding of SHH to PTCH results in internalization of both proteins to perinuclear lysosomes or endosomes^29^,^30^,^31^ the distinct SHH signals in these endocrine active cells (Fig. 4e,f) might reflect its localization to the respective vesicles and therefore might stain SHH-responding cells. In contrast, SHH might co-localize to ACTH storing vesicles in corticotropic cells (Fig. 4d).

**Figure 5:**
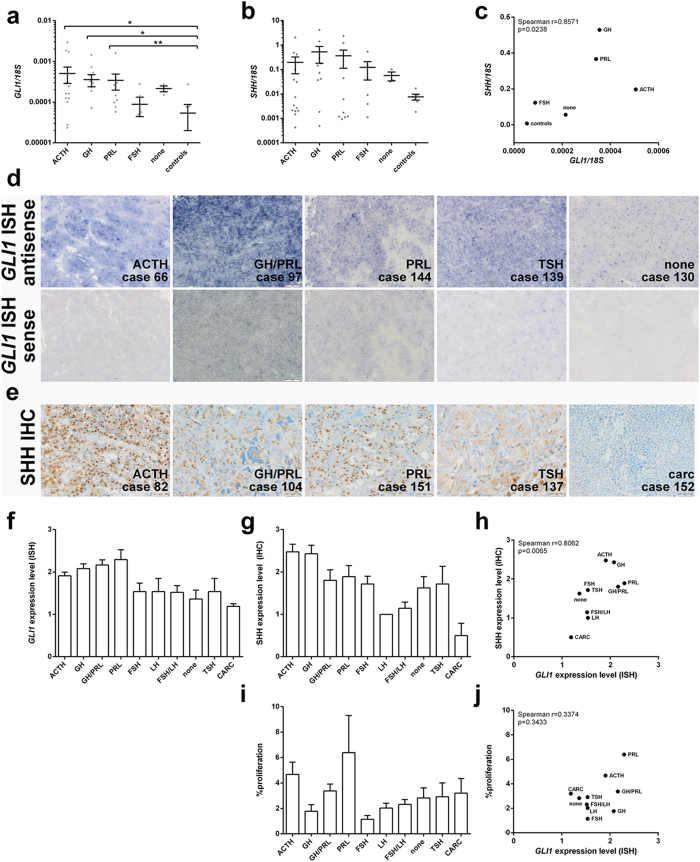
*GLI1* and *SHH*/SHH expression levels and proliferation index of human pituitary tumors. qRT-PCR-based analyses of (**a**) *GLI1* and (**b**) *SHH* expression levels and (**c**) nonparametric Spearman correlation analyses of *GLI1* and *SHH* expression levels in human PA and normal adenopituitary lobes (control) listed in Table 1. (**d**) Representative *GLI1 in situ* hybridization and (**e**) immunohistological SHH staining of pituitary tumors. (**d**) ACTH positive, mixed GH/PRL positive and PRL positive adenomas showed highest *GLI1* expression level (Score 2.5–3.5) whereas TSH positive and null cell adenomas were moderately positive for *GLI1* (Score 1–2.75). (**e**) Within the group of pituitary tumors, distribution of distinct cytoplasmic SHH immunostaining (brown color) reached high levels in ACTH positive adenomas (Score 4), moderate levels in mixed GH/PRL positive (atypical PA), PRL positive and TSH positive adenomas (Score 3). Lowest SHH staining scores were observed in pituitary carcinoma (carc) and associated metastases (Score 0). For case numbers see Table 2. (**f**) *In situ* hybridization-based analyses of *GLI1* expression levels, (**g**) immunohistology-based analyses of SHH expression levels and (**i**) percentage of Ki67^+^ proliferative tumor cells in human PA listed in Table 2 (for scoring see Material and Method section). (**h**,**j**) nonparametric Spearman correlation analyses of (**h**) *GLI1* and SHH expression levels and (**j**) *GLI1* expression levels and percentage of proliferating cells in human pituitary lesions listed in Table 2. ACTH, adreno corticotrophic hormone; GH, growth hormone; PRL, prolactin; FSH, follicle stimulating hormone; LH, luteinizing hormone; TSH, thyroid stimulating hormone; CARC, carcinoma. Mean+/−SEM, *p < 0.05, **p < 0.01. Scale bar: 20 μm.

We next quantified the *SHH* and *GLI1* transcript levels of 12 normal adenopituitaries and 48 PA by qRT-PCR (Table 1, Fig. 5a,b). *GLI1* expression levels were significantly elevated in ACTH-, GH- and PRL-expressing adenomas (p = 0.026; p = 0.035; p = 0.0059, respectively) whereas null cell adenomas and FSH-expressing tumors showed similar *GLI1* expression levels compared to normal adenopituitary lobes (Fig. 5a). Although *SHH* expression levels of the tumors did not reach significance a generally higher *SHH* expression of all PA in comparison to the controls was detected (Fig. 5b). Correlation analysis revealed that the *SHH* and *GLI1* expression levels significantly correlate on transcript level throughout the tumor collection (Fig. 5c; Spearman r-coefficient 0.8571; p = 0.0238).

Subsequently we analyzed the SHH and *GLI1* expression levels in a large cohort of 92 PA and 4 pituitary carcinomas by *GLI1 in situ* hybridization and SHH immunohistology (Table 2, Fig. 5d–g, Suppl. Figs S5 and S6). Throughout the tumor collection a strong and significant correlation of *GLI1* and SHH expression was observed (Fig. 5h; Spearman r-coefficient 0.8062; p = 0.0065). Very intense signals were detected in ACTH- and GH-producing tumors (Fig. 5d–g, Suppl. Figs S5 and S6). Prolactinomas as well as mixed GH/PRL-producing adenomas showed high *GLI1* expression levels and moderate immunoreactivity for SHH (Fig. 5d–g, Suppl. Figs S5 and S6). TSH- and FSH-expressing tumors and null cell adenomas were moderately positive for both (Fig. 5d–g, Suppl. Figs S5 and S6). LH- and FSH/LH- adenomas expressed low to moderate *GLI1* and only low SHH levels (Fig. 5d–g, Suppl. Figs S5 and S6). In pituitary carcinomas *GLI1* and SHH expression could only be hardly detected (Fig. 5d–g, Suppl. Figs S5 and S6). There was no association between HH signaling activation and proliferation rates within the tumor specimens (Fig. 5i,j).

Taken together, these data show that, in contrast to a former study^21^, not only corticotrophs but at least lactotrophs and somatotrophs of the normal human adenopituitary express SHH. Moreover, our results suggest that HH signaling activity may play a role in formation and/or maintenance of PA subtypes, especially in ACTH-, GH- or PRL-immunopositive tumors that show the highest GLI1 and SHH expression levels. However, this is pure speculation and remains to be investigated in the future.

## Discussion

Within this paper, we demonstrate that excess Hh signaling in the pituitary gland elevates the hormone expression and induces proliferative processes of Gli1^+^/Sox2^+^ and Sox9^+^ adult stem cells. However, in contrast to neural stem cells in which Hh signaling activation results in stem cell accumulation^32^ *Ptch*-depletion in the pituitary does not impact the absolute cell number but enhances the percentage of proliferating stem cells. Due to the fact that early Sox2^+^ progenitors can give rise to Sox2^+^/Sox9^+^ transit-amplifying cells^25^ which are able to generate all hormone producing cell subtypes^24^, our data thus indicate that Hh signaling activation induces proliferation and differentiation processes of Sox2^+^ cells, most likely by asymmetric divisions. This might result in maintenance of the stem cell pool and simultaneously in generation of more differentiated hormone producing daughter cells. Moreover our findings that Gli1 expression is not solely restricted to Sox2^+^ cells furthermore indicate that the Hh pathway – similarly to neural fate specification and cell cycle progression in the retina^33^ – might be involved in the function of different cellular pituitary subtypes. In fact excess Hh signaling did not induce proliferation of differentiated endocrine cells (see Fig. 3) and Acth-producing AtT-20 cells but enhanced the hormone expression in pituitary explants and *Pomc/POMC* promoter activity in AtT-20 cells. This implies that activation of Hh signaling in endocrine pituitary cells induces hormone production rather than proliferative processes. This is in contrast to Sox2^+^/Sox9^+^ pituitary stem cells, in which Hh signaling activates proliferation.

Contrary to excess Hh signaling activation due to *Ptch*-depletion a moderate pathway activity induced by Shh did not alter the proliferative capacity of primary cultured pituitary cells. In contrast, Shh induced proliferative processes in AtT-20 cells. Since this was not accompanied by elevated *Gli1* or *Gli2* expression levels we conclude that it is *impossible* to induce and study the effects of canonical Hh signaling in AtT-20 cells upon Shh treatment. The fact that Gli1 or Gli2 overexpression in AtT-20 cells also did not affect the proliferation rate of these cells supports our suggestion that activation of Hh signaling has no impact on the proliferation rate of hormone-producing pituitary cells. Beyond that these data indicate that exceeding of a certain Hh signaling threshold is necessary to induce proliferation of pituitary stem cells.

In accordance with our murine explant experiments human adenopituitaries also express *GLI1* and SHH. Moreover SHH expression pattern overlaps with hormonally active cells (e.g. ACTH-, PRL- and GH- producing cells). Similar to pituitary gland development in zebrafish^34^ these data suggest that HH signaling may also play a role in these cellular subpopulations in the adult human pituitary. Remarkably, also ACTH-, GH- or PRL-immunopositive human PA express high *SHH*/SHH and *GLI1* levels whereas TSH-, LH-, or FSH-producing tumors showed only moderate or low HH signaling activity. Previously it has been proposed that active HH signaling is restricted to ACTH-producing endocrine cells and that SHH maintains pituitary cells in a non-proliferative state. Moreover it was suggested that a *down-regulation* of HH signaling may be involved in the pathogenesis of PA^21^. In contrast to these studies we here quantified the *GLI1* transcript level which is the best indicator of HH signaling activity. Furthermore, we used a SHH antibody that detects the active N-terminal SHH fragment and thus visualizes SHH-expressing and SHH-responding cells. Finally, we revealed a significant and strong correlation of *GLI1* and *SHH*/SHH expression levels in the human PA collection (p = 0.0065 and p = 0.0238). Consequently, our results suggest that HH signaling is *active* in ACTH-, GH- and PRL-expressing cells of the adenohypophysis and in the respective PA subtypes and might indicate a role of HH signaling in the development or maintenance of PA (e.g. ACTH-, PRL-, GH-immunopositive PA). It is furthermore tempting to speculate that HH signaling activation (e.g. by *PTCH* mutations) itself drives tumor formation (e.g. in a paracrine, ligand dependent manner) and can drive hormone secretion in pituitary adenomas. However the fact that *GLI1* or *SHH*/SHH expression did not correlate with the proliferation status of PA may be explained by intratumor heterogeneity. Furthermore, Sox2 and Sox9 expression have been associated with tumor growth^35^,^36^, self-renewal of oncogene target cells, tumor initiation and invasion^37^. Moreover Sox2^+^ pituitary cells have tumor-inducing potential^36^ indicating that Sox2^+^/Sox9^+^ cells indeed play a role in PA formation. Beyond that it is interesting that pituitary carcinomas exhibit the lowest levels of HH signaling activity. These tumors are most commonly ACTH- or PRL-secreting, invasive macroadenomas that spread distant metastases. Although the analyzed case number was low, it is tempting to speculate that the conversion of a rather benign pituitary tumor (i.e. adenoma) into a metastatic tumor necessitates and correlates with downregulation of HH signaling.

To our knowledge mutations in components of the HH signaling pathway have not been reported in tumors of the pituitary. PA are the third most common intracranial tumors and the estimated prevalence in the general population is approximately 17%^38^. Surgical resection of this tumor is the primary therapy for most patients. However in a portion of patients, surgery does not result in cure^39^. Our findings suggest that the HH pathway plays a role in the pathogenesis of PA and hormone production, which however have to be verified by future experiments. If this comes true, it would be interesting to test HH pathway inhibitors (e.g. the FDA-approved HH-inhibitor vismodegib) alone or in combination with other drugs to target pituitary tumor cells (reviewed in^40^).

In summary our data demonstrate that activation of Hh signaling (e.g. by enhanced *Gli1* expression), similarly to neural fate specification and cell cycle progression in the retina^33^, induces cell type-specific cellular processes in the pituitary (e.g. hormone-release of endocrine cells and proliferation of adult pituitary stem cells). Finally, we present data which might be indicative for a role of HH signaling in the development or maintenance of PA.

## Methods

### Ethical approval and informed consent

All experimental protocols using murine or human samples were approved by the Niedersächsisches Landesamt für Verbraucherschutz und Lebensmittelsicherheit (LAVES) and by the Ethical Committee of the University of Erlangen-Nürnberg, respectively. All used methods were carried out *in accordance with* the approved guidelines of the Niedersächsisches LAVES or the Ethical Committee of the University of Erlangen-Nürnberg. For experiments involving human tissue samples written informed consent from all subjects was obtained.

### Compounds

If not otherwise stated all compounds were obtained from Sigma-Aldrich. For *in vitro* studies 5 μM cyclopamine (Toronto Research Chemicals Inc.) dissolved in ethanol (EtOH), 10 μM tamoxifen in DMSO and 1 μg/ml recombinant murine Shh-N (rShh-N; R&D systems) in HD-buffer were used. Tamoxifen solution for *in vivo* use was prepared as described (see below)^23^.

### Mice

Experiments using animals were performed in compliance with all German legal and ethical requirements. The *Rosa26CreERT2* (*CreERT2*) knock-in mouse strain (kindly provided by Dr. Anton Berns, The Netherlands Cancer Institute, Amsterdam, The Netherlands) expresses a fusion gene encoding Cre recombinase and a modified ligand-binding domain for the estrogen receptor under control of the endogenous Rosa26 promoter^41^. Generation, genotyping and CreERT2-activation of *Ptch*^*flox/flox*^*CreERT2*^+/−^ mice are described in Uhmann *et al.*^23^. Briefly, *CreERT2* mice were bred to *Ptch*^*flox/flox*^ mice to obtain *Ptch*^*flox/flox*^*CreERT2*^+/−^ mice. Eight-week-old *Ptch*^*flox/flox*^*CreERT2*^+/−^ mice were injected intraperitoneally with 1 mg tamoxifen dissolved in a 1:10 ethanol-sunflower oil emulsion^42^ on 5 consecutive days to induce the *Ptch*^*del*^ mutation (named *tPtch*^−/−^ mice) or with solvent alone. Mice with *Ptch*^*flox/flox*^*CreERT*^−/−^ genotypes were used to assess any unspecific effects of tamoxifen. After tamoxifen-mediated induction of the *Ptch*^*del*^ mutation in *Ptch*^*flox/flox*^*CreERT2*^+/−^ mice animals are named *tPtch*^−/−^ mice^23^. Solvent-injected *Ptch*^*flox/flox*^*CreERT2*^+/−^ mice or tamoxifen-injected *Ptch*^*flox/flox*^ mice served as controls. Female and male mice were used for *in vitro* and *in vivo* assays because the sex of the animals did not impact on the results.

### Buffers and media for organ culture and culture of single-cell suspensions of murine pituitaries

HD-Buffer and growth medium for organ culture and culture of single-cell suspensions of murine pituitaries consisted of 25 mM N-2-hydroxyethylpiperazine-N′-2-ethane sulfonic acid (HEPES), 137 mM NaCl, 5 mM KCl, 0.7 mM Na_2_HPO_4_, 10 mM Glucose, 1x Partricin (Biochrom) and 1% penicillin/streptomycin (PS) or Dulbeccos’s modified Eagle’s medium^++−^ (DMEM^++−^), 10% heat-inactivated FCS, 1x L-glutamine (Gibco), 1% PS, 1x Partricin, 1x minimal essential medium-vitamins (Gibco), 5 μg/ml insulin, 5 μg/ml transferrin, 60 pmol 3,3′,5-triiodo-L-thyroinine sodium salt and 20 pg/ml sodium selenite, respectively. Collagenase solution for preparing single-cell suspensions of murine pituitaries consisted of 1000 U/ml collagenase T1 (Worthington Biochem Corp.), 0.1 mg/ml trypsin inhibitor, 1 mg/ml hyaluronidase, 4 mg/ml bovine serum albumin (BSA) and 10 μg/ml DNAse II.

### Organ culture and culture of single-cell suspensions of murine pituitaries

Pituitaries of 6–8 week old *Ptch*^*flox/flox*^*CreERT2*^+/−^ and *Ptch*^*flox/flox*^ mice of both sexes were isolated and washed in HD-Buffer. For organ cultures, the glands were transferred into cell culture inserts (Falcon, Corning) in 24-well-plates filled with growth medium (see above) supplemented with tamoxifen, cyclopamine, rShh-N or the respective solvents. Media were exchanged after 2 days and pituitary glands and media were harvested for ribonucleic acid (RNA) isolation and hormone quantification, respectively, after an overall culture period of 5 days. For BrdU incorporation analyses of pituitary explants 10 μM BrdU was added to the culture medium for the last 48 h of the experiments. The vitality of the explants was verified by immunohistological stainings of active Caspase 3 (data not shown), Acth, Gh and Prl (see below) (Fig. 2).

Single pituitary cell suspensions were prepared using a collagenase solution (see above) for 1.5 h. After filtering through a 40 μm nylon cell strainer (BD Falcon) 20,000 cells were seeded for BrdU incorporation assays in 96-well-plates. After 24 h the media were changed to growth media supplemented with tamoxifen, cyclopamine or the respective solvent for 48 h. For the last 22 h of the experiments 10 μM BrdU was added to the culture medium. The experiments were conducted at least 3 times in triplicates. If not stated otherwise data represent the mean of all experiments.

### Isolation of genomic DNA and analysis of genomic recombination at the *Ptch* ^*flox*^ locus

Isolation of genomic deoxyribonucleic acid (DNA) from freshly isolated and cultured pituitary glands and quantification of the recombination efficiency at the *Ptch*^*flox*^ locus were performed as previously described^23^. For polymerase chain reaction (PCR)-based detection of the CreERT2-mediated *Ptch*^*flox*^ recombination the primer pair 5′-gcatgtgacctgcctactaattc-3′/5′-cctacttatctgatggtctgcatc-3′ was used.

### Plasmids

The Gli-binding site luciferase reporter construct (*p9xGliBS*) has been described previously^43^. The plasmid *pCR3.1 mGli1* was constructed by cloning *mGli1* complementary deoxyribonucleic acid (cDNA) from the *pcDNA3.1-His mGli1* vector^44^ (provided by Hiroshi Saraki) into the *pCR3.1* plasmid (Life Technologies). The *Gli2* expression vector *pCMV Gli2 FLAG* was provided by Chi-Chung Hui.

POMC-Prom plasmid was cloned by amplifying a 4734 bp fragment upstream of the first ATG of the human *POMC* gene sequence from genomic DNA and insertion into *pGL3-basic* (Promega GmbH). Primer sequences are available upon request. The plasmid *pRL-TK* (Promega GmbH) was used as endogenous control for the normalization of firefly luciferase activity in dual luciferase-based reporter assays.

### Cell lines and cell culture experiments

The murine PA cell line AtT-20 (ATCC; CCL-89^TM^, obtained from ATCC in July 2014) was cultured in accordance with the ATCC protocol. The Shh-N-conditioned medium (Shh-N-CM) and the respective control medium (CoM) were obtained from HEK293-Shh that express the N-terminal active fragment of Shh or HEK293 cells, respectively^45^. For gene expression analysis or BrdU incorporation assays (Roche life science) AtT-20 cells were seeded in F-12K/1% PS/2.5% FCS/15% HS at densities of 200,000 or 20,000 cells/well into 24-well- or 96-well-plates, respectively. Afterwards the cells were transfected with 2 μg plasmid DNA and RotiFect transfection reagent (Carl Roth GmbH) as indicated and/or incubated for 48 h with Shh-N-CM or CoM and cyclopamine or EtOH as indicated in the respective experiments. For the last 22 h of the experiments 10 μM BrdU was added to the culture medium. BrdU incorporation assays were performed in accordance with the manufacturer’s instructions and analyzed using a microplate reader (SynergyMX, BioTek Instruments, Inc.). For *POMC* promoter analyses 20,000 AtT-20 cells were seeded in F-12K/2.5% FCS/1.5% HS/1% PS or Shh-N-CM or CoM in 96-well-plates. Cells were transfected with 500 ng plasmid DNA as indicated in the respective experiments and 10 ng *pRL-TK* using RotiFect transfection reagent. After 48 h the activity of the *POMC* promoter reporter constructs were measured using the Dual Luciferase Assay Kit (Promega GmbH) and a microplate reader (SynergyMX). The experiments were conducted at least 3 times in triplicates. As not stated otherwise the shown data represent the mean of all experiments.

### Reverse transcription and quantitative real time-PCR-analyses (qRT-PCR)

Total RNA was extracted using TRIzol reagent (Life Technologies GmbH). cDNA synthesis, quantification of *18S* ribosomal RNA (rRNA), *Gli1* and wt *Ptch* transcripts and the standard curve method for qRT-PCR analyses were recently described^46^,^47^. Primer sequences of intron-flanking primer pairs used for relative quantification of the expression of *Ptch*^*del*^, *proopiomelanocortin (Pomc), Gh, Cga, Prl*, *Oxytocin* (*Oxt*), *luteinizing hormone* (subunit β, *Lh*β), *thyroid-stimulating hormone* (subunit β; *Tsh*β), *follicle-stimulating hormone* (subunit β; *Fsh*β), human *GLI1* and human *SHH* are listed in Supplemental Table S1. Each sample was measured in triplicates. Graphs represent the mean value of all measurements.

### Hormone determination

Acth blood serum levels were quantified using an enzyme-linked immunosorbent assay (ELISA) (Uscn Life Science Inc., BIOZOL Diagnostica). Acth concentrations of supernatants of cultured pituitary were quantified using the IMMULITE^®^ 2000 Immunoassay System (Siemens AG).

### *In situ* hybridization, immunohistological and immunofluorescent stainings of cultured murine pituitary gland

Cultured murine pituitary glands were fixed in 4% PFA, embedded in paraffin and sectioned. The sections were stained with hematoxylin and eosin (H&E) for histopathological analyses or were analyzed by *in situ* hybridization, immunohistological or immunofluorescent stainings. The *in situ* hybridization procedure and the probes for detecting wt *Ptch* and whole *Ptch* transcripts have been described previously^48^. Immunohistological and immunofluorescent stainings were conducted by boiling the sections in 10 mM citric acid, pH 6.0 (which also allows for specific staining with anti-BrdU antibodies)^49^ and staining with the antibodies listed in Supplemental Table S2. The specificity of the antibodies was verified by showing that antibodies against Pomc, Acth, Prl and Gh detected selective populations of cells in the anterior pituitary gland, antibodies against Sox2 and Sox9 detected selective populations of cells in the marginal zone and in the anterior pituitary gland. None of the antibodies detected antigens in the posterior lobe of the pituitary. The specificity of the antibody against Gli1 and Caspase 3 was verified by specific staining of murine basal cell carcinoma. The specificity of the antibody against BrdU was verified by showing that the antibody did not detect antigens in pituitary explants without BrdU-treatment.

Negative controls were carried out by incubation in the absence of the primary antibody and always yielded negative results.

### Human pituitary tissue samples

Surgical specimens from 142 different patients with sellar lesions (n = 145) were retrieved from the Department of Neuropathology of the University Hospital of Erlangen. Each tumor specimen was classified and graded according to the currently valid version of the World Health Organization classification system of tumors of endocrine organs^22^. Twelve normal pituitary tissues and five different tumor subtypes (n = 48) were analyzed by qRT-PCR for relative *GLI1* and *SHH* expression level (data for each case are summarized in Table 1). Fifteen normal pituitary tissues and ten different tumor subtypes (n = 96; data for each case are summarized in Table 2) were analyzed by specific histological stainings (*GLI1 in situ* hybridization, SHH immunohistological stainings). Tumors were grouped according to clinical symptoms and hormone release as following: 1) ACTH producing adenomas with and without clinical signs of Cushing’s disease (qRT-PCR: 7 male, 9 female, median age 49.7 years; specific histological stainings: 23 tumors from 22 different patients; 8 male,14 female, median age 42.8 years); 2) adenomas from patients with clinical signs of acromegaly and immunohistochemical expression of GH (qRT-PCR: 6 male, 6 female, median age 39.4 years; specific histological stainings: 6 male, 8 female, median age 45.2 years); 3) mixed adenomas from patients with clinical signs of acromegaly and combined immunohistochemical expression of GH and PRL (specific histological stainings: 4 male, 6 female, median age 31.8 years); 4) clinically nonfunctioning tumors with expression of FSH (qRT-PCR: 4 male, 2 female, median age 54.1 years; specific histological stainings: 5 male, 2 female, median age 51.7 years); 5) clinically nonfunctioning adenomas with expression of LH (specific histological stainings: 4 male, 3 female, median age 61.4 years); 6) clinically nonfunctioning tumors with combined expression of FSH and LH (specific histological stainings: 6 male, 1 female, median age 58.3 years); 7) nonfunctioning adenomas without detectable hormone expression (qRT-PCR: 2 male, 1 female, median age 49.0 years; null cell adenomas; specific histological stainings: 5 male, 3 female, median age 46.8 years); 8) TSH producing adenomas (specific histological stainings: 2 male, 5 female, median age 38 years); 9) PRL producing adenomas (qRT-PCR: 3 male, 8 female, median age 46.7 years; specific histological stainings: 6 male, 3 female, median age 39.4 years); 10) pituitary carcinomas (specific histological stainings: 4 tumors and/or metastases from 3 different patients; 2 male, 1 female, median age 39 years). Data for each case included in the study are shown in Table 1 (qRT-PCR) and Table 2 (specific histological stainings).

Normal pituitary tissue samples (n = 27) were acquired from patients with sellar exploration in cases of magnetic resonance imaging negative microadenomas and Rathke’s cleft cysts. The latter specimens showed a regular lobulated reticulin fiber network typically observed in the adenohypophysis and IHC confirmed the regular spectrum of hormone production.

The study concept was approved by the Ethical Committee of the University of Erlangen-Nürnberg.

### IHC procedure and evaluation on human samples

IHC was performed as described using a semiautomated benchmark apparatus (Nexes; Ventana, Illkirch, France) and the Ventana DAB staining system^50^. Positive and negative controls were used to validate the staining of the primary antibodies listed in Supplemental Table S3. The specificity of the antibodies was tested by staining human adenopituitaries showing selective populations of cells only. The specificity of the anti-SHH antibody was verified by staining human pancreas carcinoma and intestine. Negative controls were carried out by incubation in the absence of the primary antibody and always yielded negative results.

The intensity of SHH staining was assessed semi-quantitatively blinded to any diagnosis concerning the hormonal status of patients (D.S. and R.B.) and grouped into five different categories (0 = no SHH expression; 1 = very low intensity, 2 = moderate intensity, 3 = strong intensity, 4 = highest intensity of all). In cases without detectable SHH expression, the IHC was repeated to verify the result. The proliferation rate of each tumor was assessed quantitatively (D.S.) by counting a minimum of 1,000 tumor cell nuclei in a region with the highest amount of distinct positive Ki76^+^ cell nuclei.

### *In situ* hybridization and evaluation on human samples

*In situ* hybridization was conducted as described^48^,^51^ using antisense and sense (negative control) riboprobes reversely transcribed from a *pBS-hGLI1* plasmids containing the *hGLI1* cDNA (GenBank: X07384.1) (provided by Mark Wijgerde).

The intensity of *GLI1 in situ* hybridization was assessed semi-quantitatively blinded to any diagnosis concerning the hormonal status of patients (A.U., D.S. and R.B.) and grouped into 7 different categories (0 = no expression; 0.5 = very low intensity, 1 = low intensity, 2 = intermediate intensity, 2.5 = intermediate to strong intensity, 3 = strong intensity, 3.5 = highest intensity of all).

### Statistical analyses

Statistical analyses were conducted using the software GraphPadPrism 6 (GraphPad Software Inc.). Correlation or significance of *GLI1* and *SHH*/SHH expression in human PA was tested by a nonparametric Spearman correlation test or unpaired nonparametric Mann-Whitney test, respectively. Statistical significance of the recombination efficiency at the *Ptch*^*flox*^ locus, the gene expression in pituitary glands and human PA, the Acth blood serum levels and Acth concentration of media were tested using Holm-Sidak’s multiple comparison test (unpaired t test). Statistical significance of gene expression levels of *ex vivo* cultured pituitaries was tested after outliers correction using ROUT method (Q = 1%) and testing the Gaussian distribution by D’Agustino and Pearson omnibus normality test followed by an one-way-ANOVA (analysis of variance; Holm-Sidak’s multiple comparison test). Statistical significance of proliferative Sox2^+^ or Sox9^+^ cells, of BrdU incorporation assays of *in vitro* cultured pituitary cells or AtT-20 cells, of dual-luciferase assays and expression analyses of AtT-20 cells were tested using nonparametric Mann-Whitney test, Dunn’s multiple comparison test or Kruskal-Wallis test, respectively.

## Additional Information

**How to cite this article**: Pyczek, J. *et al.* Hedgehog signaling activation induces stem cell proliferation and hormone release in the adult pituitary gland. *Sci. Rep.* **6**, 24928; doi: 10.1038/srep24928 (2016).

## Supplementary Material

Supplementary Dataset S1

## Figures and Tables

**Table 1:** Relative quantification of *GLI1* and *SHH* expression levels in human pituitary adenomas and adenohypophyses.

patient no.	age	sex	IHC	*GLI1*/*18S* (mean ± SEM × 10^−4^)		*SHH/18S* (mean ± SEM × 10^−4^)	
**1**	30	m	ACTH	0.12	0.003	0.00	0.000
**2**	44	f	ACTH	0.00	0.000	0.00	0.000
**3**	47	f	ACTH	0.16	0.053	2.13	0.317
**4**	49	m	ACTH	0.28	0.091	2.67	0.442
**5**	36	f	ACTH	1.86	0.509	1.46	0.162
**6**	62	f	ACTH	0.00	0.000	3.41	0.790
**7**	39	f	ACTH	2.95	0.290	489.94	33.993
**8**	64	f	ACTH	0.02	0.002	31.06	5.662
**9**	69	m	ACTH	1.73	0.041	38.41	4.019
**10**	33	f	ACTH	0.03	0.002	0.42	0.034
**11**	51	m	ACTH	0.31	0.040	2064.58	139.327
**12**	50	f	ACTH	0.16	0.011	1.99	0.130
**13**	65	m	ACTH	0.24	0.040	412.76	69.239
**14**	21	f	ACTH	0.00	0.000	2.01	0.353
**15**	66	m	ACTH	0.12	0.019	101.15	4.176
**16**	58	m	ACTH	0.10	0.018	7.42	0.214
**17**	53	f	GH	0.00	0.000	0.00	0.000
**18**	40	m	GH	0.33	0.016	0.00	0.000
**19**	45	m	GH	0.23	0.041	1.85	0.466
**20**	32	f	GH	0.07	0.003	146.92	12.587
**21**	33	m	GH	0.53	0.081	1415.44	53.226
**22**	57	f	GH	0.72	0.085	37.07	2.046
**23**	36	m	GH	0.00	0.000	288.98	9.869
**24**	65	f	GH	0.45	0.029	4167.47	7.086
**25**	39	m	GH	0.19	0.015	71.83	6.497
**26**	25	f	GH	0.00	0.000	0.48	0.028
**27**	35	m	GH	1.44	0.082	178.02	6.736
**28**	41	f	GH	0.30	0.010	41.33	2.662
**29**	20	f	PRL	0.15	0.014	1235.95	66.472
**30**	69	m	PRL	0.06	0.015	1.11	0.099
**31**	18	m	PRL	1.59	0.118	1.21	0.202
**32**	45	f	PRL	0.26	0.041	2372.95	188.663
**33**	34	f	PRL	0.30	0.028	0.00	0.000
**34**	55	f	PRL	0.42	0.024	0.93	0.048
**35**	53	f	PRL	0.10	0.004	1.19	0.292
**36**	34	f	PRL	0.11	0.021	6.10	0.703
**37**	47	f	PRL	0.08	0.007	44.43	2.871
**38**	50	m	PRL	0.35	0.028	1.72	0.270
**39**	47	f	PRL	0.43	0.102	1937.14	243.703
**40**	45	m	FSH	0.00	0.000	1.11	0.122
**41**	71	f	FSH	0.00	0.000	142.39	16.203
**42**	76	m	FSH	0.28	0.064	540.21	42.347
**43**	47	m	FSH	0.14	0.013	41.89	0.255
**44**	61	f	FSH	0.05	0.002	9.01	0.338
**45**	44	m	FSH	0.06	0.008	3.88	1.116
**46**	49	m	none	0.15	0.011	31.60	0.694
**47**	42	f	none	0.27	0.072	100.81	3.977
**48**	77	m	none	0.22	0.016	36.44	0.820
**49**	80	f	control	0.08	0.003	2.10	0.739
**50**	59	m	control	0.08	0.025	0.69	0.068
**51**	42	m	control	0.00	0.000	34.70	1.329
**52**	89	f	control	0.00	0.000	0.89	0.186
**53**	54	m	control	0.00	0.000	n.d.	n.d.
**54**	72	m	control	0.27	0.004	4.96	0.157
**55**	87	f	control	0.00	0.000	0.00	0.000
**56**	54	f	control	0.00	0.000	1.83	0.032
**57**	45	f	control	n.d.	n.d.	5.82	0.100
**58**	91	f	control	n.d.	n.d.	16.80	2.175
**59**	74	m	control	n.d.	n.d.	7.42	0.387
**60**	62	f	control	n.d.	n.d.	9.19	0.535

Given are age and sex of patients with hormone-active or -inactive pituitary adenomas and of donors of adenohypophysis control tissue (control). The *GLI1* and *SHH* expression levels of the samples were quantified by qRT-PCR as described in the Material and Methods section. m, male; f, female; ACTH, adrenocorticotrophic hormone; GH, growth hormone; PRL, prolactin; FSH, follicle stimulating hormone. n.d. not determined.

**Table 2:** Expression of *GLI1* and SHH in human pituitary adenomas.

patient no.	age	sex	IHC	*GLI1*	SHH	% proliferation
**61**	39	m	ACTH	2.00	2.00	1.5
**62**	34	f	ACTH	2.00	2.00	2.3
**63**	39	f	ACTH	1.75	3.00	6.6
**64**	47	f	ACTH	1.75	3.00	0.8
**65**	20	f	ACTH	2.25	2.00	7.0
**66**	27	f	ACTH#	2.00	2.00	1.3
**66**	21	f	ACTH#	2.50	1.00	5.4
**67**	29	f	ACTH	2.00	4.00	3.0
**68**	21	f	ACTH	1.75	n.d.	n.d.
**69**	46	m	ACTH	0.75	2.00	7.5
**70**	63	f	ACTH*	2.25	3.00	11.3
**71**	49	f	ACTH	2.00	2.00	15.9
**72**	69	f	ACTH	1.63	4.00	8.2
**73**	38	f	ACTH	2.00	2.00	7.4
**74**	58	f	ACTH	1.75	2.00	1.4
**75**	55	m	ACTH	2.00	3.00	1.5
**76**	25	m	ACTH	1.00	n.d.	1.4
**77**	37	m	ACTH	2.25	2.00	0.5
**78**	32	f	ACTH	1.75	2.00	2.5
**79**	31	f	ACTH	2.00	3.00	14.0
**80**	43	m	ACTH	1.75	2.00	1.4
**81**	68	m	ACTH	2.00	2.00	0.9
**82**	51	m	ACTH	2.75	4.00	1.0
**83**	36	m	GH	2.25	3.00	1.1
**84**	50	f	GH	2.25	3.00	6.5
**85**	34	m	GH	1.50	1.00	2.4
**86**	34	f	GH	2.50	1.00	5.8
**87**	52	f	GH	2.00	2.00	0.3
**88**	70	m	GH	2.25	2.00	1.9
**89**	30	f	GH	2.25	2.00	1.3
**90**	44	f	GH	2.25	3.00	1.4
**91**	46	m	GH	n.d.	2.00	0.7
**92**	37	f	GH	2.50	3.00	0.3
**93**	72	f	GH	2.00	3.00	0.8
**94**	37	f	GH	2.00	3.00	1.2
**95**	46	m	GH	1.00	3.00	0.5
**96**	45	m	GH	2.25	3.00	0.6
**97**	33	f	GH/PRL	2.75	2.00	2.6
**98**	33	m	GH/PRL	2.50	2.00	2.7
**99**	11	f	GH/PRL	2.00	1.00	3.0
**100**	15	f	GH/PRL	2.00	3.00	5.8
**101**	61	f	GH/PRL	2.00	1.00	0.9
**102**	54	f	GH/PRL	2.00	2.00	1.6
**103**	23	m	GH/PRL	2.25	2.00	2.3
**104**	29	m	GH/PRL*	2.25	3.00	5.8
**105**	33	m	GH/PRL*	1.38	1.00	4.2
**106**	26	f	GH/PRL*	2.50	1.00	4.8
**107**	66	m	FSH	1.00	2.00	1.2
**108**	41	m	FSH	1.00	1.00	2.8
**109**	46	m	FSH	1.75	2.00	1.2
**110**	46	f	FSH	2.00	2.00	0.4
**111**	38	f	FSH	2.25	1.00	0.5
**112**	57	m	FSH	1.00	2.00	1.0
**113**	68	m	FSH	1.75	2.00	0.9
**114**	67	f	LH	0.00	1.00	1.8
**115**	74	m	LH	2.50	1.00	0.6
**116**	51	m	LH	1.50	1.00	2.5
**117**	65	m	LH	2.00	1.00	3.3
**118**	65	f	LH	1.00	1.00	2.4
**119**	47	f	LH	2.00	1.00	2.8
**120**	61	m	LH	1.75	1.00	0.8
**121**	66	f	FSH/LH	1.75	1.00	1.7
**122**	43	m	FSH/LH	2.00	1.00	1.0
**123**	59	m	FSH/LH	1.00	1.00	1.4
**124**	57	m	FSH/LH	1.00	1.00	2.3
**125**	75	m	FSH/LH	1.50	1.00	3.2
**126**	48	m	FSH/LH	2.00	2.00	3.9
**127**	60	m	FSH/LH	1.38	1.00	2.7
**128**	50	m	none	2.00	2.00	5.0
**129**	67	m	none	2.50	1.00	1.3
**130**	19	m	none	1.00	2.00	3.6
**131**	65	f	none	1.00	1.00	0.8
**132**	57	f	none	1.00	1.00	0.6
**133**	41	f	none	0.75	1.00	1.0
**134**	23	m	none*	1.38	3.00	6.5
**135**	52	m	none*	1.25	2.00	3.8
**136**	69	f	TSH	2.50	2.00	2.8
**137**	19	m	TSH	1.00	3.00	9.0
**138**	31	f	TSH	1.00	2.00	3.7
**139**	30	f	TSH	2.75	1.00	0.7
**140**	35	m	TSH	1.50	0.00	0.8
**141**	51	f	TSH	1.50	3.00	2.0
**142**	31	f	TSH	0.50	1.00	1.4
**143**	32	m	PRL	2.50	1.00	0.7
**144**	27	m	PRL	3.25	1.00	1.6
**145**	43	m	PRL	2.00	2.00	3.2
**146**	26	f	PRL	2.50	2.00	1.1
**147**	39	f	PRL	2.50	3.00	1.6
**148**	18	f	PRL	0.75	2.00	6.5
**149**	52	m	PRL	2.75	2.00	3.4
**150**	40	m	PRL*	2.38	1.00	11.6
**151**	78	m	PRL*	2.00	3.00	27.8
**152**	23	m	ACTH$,§	1.25	0.00	5.5
**152**	24	m	ACTH$,§	1.00	1.00	2.1
**153**	49	m	PRL$	1.25	0.00	20.5
**154**	60	f	PRL$	1.25	1.00	2.0

Given are age and sex of patients with hormone-active or -inactive pituitary tumors. The *GLI1* and SHH expression levels and the percentage of proliferative tumor cells were scored as described in the Material and Methods section. m, male; f, female; ACTH, adrenocorticotrophic hormone; GH, growth hormone; PRL, prolactin; FSH, follicle stimulating hormone; LH, luteinizing hormone; TSH, thyroid stimulating hormone. *atypic adenoma; ^$^carcinoma; ^#^same patient; ^§^same patient; n.d. not determined.

## References

[b1] Hooper J. E. & Scott M. P. Communicating with Hedgehogs. Nat Rev Mol Cell Biol 6, 306–317 (2005). 15803137 10.1038/nrm1622

[b2] Machold R. *et al.* Sonic hedgehog is required for progenitor cell maintenance in telencephalic stem cell niches. Neuron 39, 937–950 (2003). 12971894 10.1016/s0896-6273(03)00561-0

[b3] Shin K. *et al.* Hedgehog/Wnt feedback supports regenerative proliferation of epithelial stem cells in bladder. Nature 472, 110–114, 10.1038/nature09851 (2011). 21389986 PMC3676169

[b4] Roessler E. *et al.* Loss-of-function mutations in the human GLI2 gene are associated with pituitary anomalies and holoprosencephaly-like features. Proc Natl Acad Sci USA 100, 13424–13429 (2003). 14581620 10.1073/pnas.2235734100 PMC263830

[b5] Kimura S. *et al.* The T/ebp null mouse: thyroid-specific enhancer-binding protein is essential for the organogenesis of the thyroid, lung, ventral forebrain, and pituitary. Genes Dev 10, 60–69 (1996). 8557195 10.1101/gad.10.1.60

[b6] Pabst O., Herbrand H., Takuma N. & Arnold H. H. NKX2 gene expression in neuroectoderm but not in mesendodermally derived structures depends on sonic hedgehog in mouse embryos. Dev Genes Evol 210, 47–50 (2000). 10603087 10.1007/pl00008188

[b7] Franca M. M. *et al.* Novel heterozygous nonsense GLI2 mutations in patients with hypopituitarism and ectopic posterior pituitary lobe without holoprosencephaly. J Clin Endocrinol Metab 95, E384–391 (2010). 20685856 10.1210/jc.2010-1050

[b8] Flemming G. M. *et al.* Functional characterization of a heterozygous GLI2 missense mutation in patients with multiple pituitary hormone deficiency. J Clin Endocrinol Metab 98, E567–575, 10.1210/jc.2012-3224 (2013). 23408573 PMC3590478

[b9] Treier M. *et al.* Hedgehog signaling is required for pituitary gland development. Development 128, 377–386 (2001). 11152636 10.1242/dev.128.3.377

[b10] Bale S. J., Amos C. I., Parry D. M. & Bale A. E. Relationship between head circumference and height in normal adults and in the nevoid basal cell carcinoma syndrome and neurofibromatosis type I. Am J Med Genet 40, 206–210, 10.1002/ajmg.1320400217 (1991). 1910262

[b11] Bale A. E., Gailani M. R. & Leffell D. J. Nevoid basal cell carcinoma syndrome. J Invest Dermatol 103, 126S–130S (1994). 7963674 10.1111/1523-1747.ep12399438

[b12] Wicking C. & Bale A. E. Molecular basis of the nevoid basal cell carcinoma syndrome. Curr Opin Pediatr 9, 630–635 (1997). 9425597 10.1097/00008480-199712000-00013

[b13] Lo Muzio L. *et al.* Nevoid basal cell carcinoma syndrome. Clinical findings in 37 Italian affected individuals. Clin Genet 55, 34–40 (1999). 10066029 10.1034/j.1399-0004.1999.550106.x

[b14] Hahn H. *et al.* Rhabdomyosarcomas and radiation hypersensitivity in a mouse model of Gorlin syndrome. Nature Med 4, 619–622 (1998). 9585239 10.1038/nm0598-619

[b15] Cramer H. & Niederdellmann H. Cerebral gigantism associated with jaw cyst basal cell naevoid syndrome in two families. Arch Psychiatr Nervenkr (1970) 233, 111–124 (1983). 6882181 10.1007/BF00343432

[b16] Kahn L. B. & Gordon W. The basal cell naevus syndrome–report of a case. S Afr Med J 41, 832–835 (1967). 6060321

[b17] Codish S. D., Kraszeski J. & Pratt K. CNS developmental anomaly in the basal cell nevus syndrome: another congenital neurocutaneous syndrome? Neuropadiatrie 4, 338–343, 10.1055/s-0028-1091750 (1973). 4800494

[b18] Marcos M. V., Querol X., Armengol A., Hierro F. R. & Cruz M. [Basal cell nevus syndrome and gigantism]. An Esp Pediatr 16, 513–519 (1982). 7125403

[b19] Kimonis V. E. *et al.* Clinical manifestations in 105 persons with nevoid basal cell carcinoma syndrome. Am J Med Genet 69, 299–308 (1997). 9096761

[b20] Vila G. *et al.* Sonic hedgehog regulates CRH signal transduction in the adult pituitary. Faseb J 19, 281–283 (2005). 15572433 10.1096/fj.04-2138fje

[b21] Vila G. *et al.* Expression and function of sonic hedgehog pathway components in pituitary adenomas: evidence for a direct role in hormone secretion and cell proliferation. J Clin Endocrinol Metab 90, 6687–6694 (2005). 16159933 10.1210/jc.2005-1014

[b22] The International Agency for Research on Cancer Pathology and Genetics of Tumours of Endocrine Organs (IARC WHO Classification of Tumours). Vol. 1 (eds DeLellis R. A. *et al.*) (IARC Press, Lyon, France, 2004).

[b23] Uhmann A. *et al.* The Hedgehog receptor Patched controls lymphoid lineage commitment. Blood 110, 1814–1823 (2007). 17536012 10.1182/blood-2007-02-075648

[b24] Rizzoti K., Akiyama H. & Lovell-Badge R. Mobilized adult pituitary stem cells contribute to endocrine regeneration in response to physiological demand. Cell Stem Cell 13, 419–432, 10.1016/j.stem.2013.07.006 (2013). 24094323 PMC3793864

[b25] Fauquier T., Rizzoti K., Dattani M., Lovell-Badge R. & Robinson I. C. SOX2-expressing progenitor cells generate all of the major cell types in the adult mouse pituitary gland. Proc Natl Acad Sci USA 105, 2907–2912, 10.1073/pnas.0707886105 (2008). 18287078 PMC2268558

[b26] Heaney A. P., Fernando M., Yong W. H. & Melmed S. Functional PPAR-gamma receptor is a novel therapeutic target for ACTH-secreting pituitary adenomas. Nat Med 8, 1281–1287, 10.1038/nm784 (2002). 12379847

[b27] Shan B. *et al.* Curcumin suppresses HIF1A synthesis and VEGFA release in pituitary adenomas. J Endocrinol 214, 389–398, 10.1530/JOE-12-0207 (2012). 22739211

[b28] Lampichler K. *et al.* The role of proto-oncogene GLI1 in pituitary adenoma formation and cell survival regulation. Endocr Relat Cancer 22, 793–803, 10.1530/ERC-15-0109 (2015). 26219678

[b29] Incardona J. P. *et al.* Receptor-mediated endocytosis of soluble and membrane-tethered Sonic hedgehog by Patched-1. Proc Natl Acad Sci USA 97, 12044–12049, 10.1073/pnas.220251997 (2000). 11027307 PMC17291

[b30] Yue S. *et al.* Requirement of Smurf-mediated endocytosis of Patched1 in sonic hedgehog signal reception. Elife 3, 10.7554/eLife.02555 (2014). PMC4080449 24925320

[b31] Mastronardi F. G., Dimitroulakos J., Kamel-Reid S. & Manoukian A. S. Co-localization of patched and activated sonic hedgehog to lysosomes in neurons. Neuroreport 11, 581–585 (2000). 10718318 10.1097/00001756-200002280-00030

[b32] Ferent J. *et al.* Genetic activation of Hedgehog signaling unbalances the rate of neural stem cell renewal by increasing symmetric divisions. Stem Cell Reports 3, 312–323, 10.1016/j.stemcr.2014.05.016 (2014). 25254344 PMC4175546

[b33] Sakagami K., Gan L. & Yang X. J. Distinct effects of Hedgehog signaling on neuronal fate specification and cell cycle progression in the embryonic mouse retina. J Neurosci 29, 6932–6944, 10.1523/JNEUROSCI.0289-09.2009 (2009). 19474320 PMC2715855

[b34] Devine C. A. *et al.* A dynamic Gli code interprets Hh signals to regulate induction, patterning, and endocrine cell specification in the zebrafish pituitary. Dev Biol 326, 143–154, 10.1016/j.ydbio.2008.11.006 (2009). 19056374

[b35] Vanner R. J. *et al.* Quiescent sox2(+) cells drive hierarchical growth and relapse in sonic hedgehog subgroup medulloblastoma. Cancer Cell 26, 33–47, 10.1016/j.ccr.2014.05.005 (2014). 24954133 PMC4441014

[b36] Andoniadou C. L. *et al.* Sox2(+) stem/progenitor cells in the adult mouse pituitary support organ homeostasis and have tumor-inducing potential. Cell Stem Cell 13, 433–445, 10.1016/j.stem.2013.07.004 (2013). 24094324

[b37] Larsimont J. C. *et al.* Sox9 Controls Self-Renewal of Oncogene Targeted Cells and Links Tumor Initiation and Invasion. Cell Stem Cell 17, 60–73, 10.1016/j.stem.2015.05.008 (2015). 26095047

[b38] Ezzat S. *et al.* The prevalence of pituitary adenomas: a systematic review. Cancer 101, 613–619, 10.1002/cncr.20412 (2004). 15274075

[b39] Heaney A. Management of aggressive pituitary adenomas and pituitary carcinomas. J Neurooncol 117, 459–468, 10.1007/s11060-014-1413-6 (2014). 24584748

[b40] Gupta S., Takebe N. & Lorusso P. Targeting the Hedgehog pathway in cancer. Ther Adv Med Oncol 2, 237–250, 10.1177/1758834010366430 (2010). 21789137 PMC3126020

[b41] Hameyer D. *et al.* Toxicity of ligand-dependent Cre recombinases and generation of a conditional Cre deleter mouse allowing mosaic recombination in peripheral tissues. Physiol Genomics 31, 32–41 (2007). 17456738 10.1152/physiolgenomics.00019.2007

[b42] Imai T., Jiang M., Chambon P. & Metzger D. Impaired adipogenesis and lipolysis in the mouse upon selective ablation of the retinoid X receptor alpha mediated by a tamoxifen-inducible chimeric Cre recombinase (Cre-ERT2) in adipocytes. Proc Natl Acad Sci USA 98, 224–228 (2001). 11134524 10.1073/pnas.011528898 PMC14572

[b43] Beer C., Buhr P., Hahn H., Laubner D. & Wirth M. Gene expression analysis of murine cells producing amphotropic mouse leukaemia virus at a cultivation temperature of 32 and 37 degrees C. J Gen Virol 84, 1677–1686 (2003). 12810861 10.1099/vir.0.18871-0

[b44] Sasaki H., Nishizaki Y., Hui C., Nakafuku M. & Kondoh H. Regulation of Gli2 and Gli3 activities by an amino-terminal repression domain: implication of Gli2 and Gli3 as primary mediators of Shh signaling. Development 126, 3915–3924 (1999). 10433919 10.1242/dev.126.17.3915

[b45] Chen J. K., Taipale J., Young K. E., Maiti T. & Beachy P. A. Small molecule modulation of Smoothened activity. Proc Natl Acad Sci USA 99, 14071–14076 (2002). 12391318 10.1073/pnas.182542899 PMC137838

[b46] Ecke I. *et al.* Cyclopamine treatment of full-blown Hh/Ptch-associated RMS partially inhibits Hh/Ptch signaling, but not tumor growth. Mol Carcinog 47, 361–372 (2008). 17963245 10.1002/mc.20394

[b47] Nitzki F. *et al.* Tumor stroma-derived Wnt5a induces differentiation of basal cell carcinoma of Ptch mutant mice via CaMKII. Cancer Res 70, 2739–2748 (2010). 20233865 10.1158/0008-5472.CAN-09-3743

[b48] Zibat A. *et al.* Time-point and dosage of gene inactivation determine the tumor spectrum in conditional Ptch knockouts. Carcinogenesis 30, 918–926 (2009). 19321799 10.1093/carcin/bgp068

[b49] Tang X., Falls D. L., Li X., Lane T. & Luskin M. B. Antigen-retrieval procedure for bromodeoxyuridine immunolabeling with concurrent labeling of nuclear DNA and antigens damaged by HCl pretreatment. J Neurosci 27, 5837–5844, 10.1523/JNEUROSCI.5048-06.2007 (2007). 17537952 PMC6672250

[b50] Buslei R. *et al.* Activation and regulation of endogenous retroviral genes in the human pituitary gland and related endocrine tumors. Neuropathol Appl Neurobiol, 10.1111/nan.12136 (2014). 24635849

[b51] Wijgerde M., Ooms M., Hoogerbrugge J. W. & Grootegoed J. A. Hedgehog signaling in mouse ovary: Indian hedgehog and desert hedgehog from granulosa cells induce target gene expression in developing theca cells. Endocrinology 146, 3558–3566, 10.1210/en.2005-0311 (2005). 15878962

