# Supplementary material for: Hedgehog signaling activation induces stem cell proliferation and hormone release in the adult pituitary gland
Source: Sci Rep. 2016 Apr 25;6:24928. doi: 10.1038/srep24928 (PMC4842994; doi:10.1038/srep24928)
Supplement: Supplementary Dataset S1 [file srep24928-s1.doc]

**Supplemental information to the manuscript:**

**Hedgehog signaling activation induces stem cell proliferation and hormone release in the adult pituitary gland**

Joanna Pyczek^1^, Rolf Buslei^2^, David Schult^2^, Annett Hölsken^2^, Michael Buchfelder^3^, Ina Heß^1^, Heidi Hahn^1^ and Anja Uhmann^1*^

**Supplemental Table S1: Sequences of intron-flanking primer** pairs used for relative quantification of gene expression.

| **transcript** | **forward primer** | **reverse primer** |
| --- | --- | --- |
| *Ptch^del^* ^(*)^ | 5’-tgc aaa cca tgt ttc cag tta-3’ | 5’-ttg ggg cga cac ttt gat-3’ |
| *Pomc* | 5’-cag acc tcc ata gat gtg tgg ag-3’ | 5’-gcg gaa gtg acc cat gac gta c-3’ |
| *Gh* | 5’-aag agg aca tcc agg ctc t-3’ | 5’-cgt cgt cgc tgc gca tgt t -3’ |
| *Cga* | 5’-gtc att ctg gtc atg ctg tcc atg-3’ | 5’-gat atg ccc tgg aga agc aac agc-3’ |
| *Prl* | 5’-gag agc tgt ttg acc gtg tgg-3’ | 5’-gat gac ctt gac cat aaa ctc ac-3’ |
| *Oxt* | 5’-gca aga ggg ctg tgc tgg acc t-3’ | 5’-gtc cgc gca gca gat gct tgg t-3’ |
| *Lhb* | 5’-cca agg tag gga agg tat caa gaa tgg-3’ | 5’-cag gcc ggc aca gtg gcc gaa gg-3’ |
| *Tshb* | 5’-tgt gct ggg tat tgt atg aca cgg-3’ | 5’-att cgt tct att cca ggt aaa cac a-3’ |
| *Fshb* | 5’-agc atc aat acc act tgg tgt gcg-3’ | 5’-agg tac ata ctt tct ggg tat tgg gc-3’ |
| *GLI1* | 5’-agc tac atc aac tcc ggc ca-3’ | 5’-gct gcg gcg ttc aag aga-3’ |
| *SHH* | 5’-gat gac tca gag gtg taa gga c-3’ | 5’-cct cgt tag tgc aga gac tcc-3’ |

(*) For quantification of *Ptch^del^* transcripts a probe-based qRT-PCR assay using a 5’-FAM-acc acc tcc acg taa gtc ctc t-BHQ1 probe was used.

**Supplemental Table S2: Antibodies used for immunohistochemical and immunofluorescent stainings of murine tissues.**

| **protein** | **host** | **clone** | **distributor** | **dilution** | **reference** |
| --- | --- | --- | --- | --- | --- |
| Acth | rabbit | AFP-156102789 | NHPP | 1:1000 | [^1^](#_ENREF_1) |
| active Caspase 3 | rabbit | AF835 | R&D Systems | 1:500 | [^2^](#_ENREF_2) |
| BrdU | rat | Ab6326 | Abcam | 1:100 | [^3^](#_ENREF_3) |
| Gh | rabbit | AFP-5641801 | NHPP | 1:1000 | [^4^](#_ENREF_4) |
| Gli1 | rabbit | 100-401-223 | Rockland Immunochemicals | 1:100 | [^5^](#_ENREF_5) |
| Pomc | goat | NB100-1533 | Novus Biologicals | 1:200 | [^6^](#_ENREF_6) |
| Prl | rabbit | AFP-879151 | NHPP | 1:1000 | [^4^](#_ENREF_4) |
| Sox2 | rabbit | EPR3131 | Abcam | 1:100 | [^7^](#_ENREF_7) |
| Sox2 | rat | Btjce | eBioscience | 1:100 | [^8^](#_ENREF_8) |
| Sox9 | rabbit | AB5535 | Millipore | 1:2500 | [^4^](#_ENREF_4) |

Immunohistological staining was visualized by Dako EnVision Polyclonal rabbit/mouse HRP (K5007) and AEC or DAB chromogene. For double immunofluorescent stainings secondary FITC-labeled goat anti-rat (1:200), Cy3-labeled goat anti-rabbit (1:400) or donkey anti goat FITC-labeled antibodies (1:200) (Dianova) were used. Nuclei were counterstained with ProLong Gold antifade reagent with DAPI (molecular probes).

**Supplemental Table S3: Antibodies used for immunohistochemical and immunofluorescent stainings of** human tissues.

| **protein** | **host** | **clone** | **distributor** | **dilution** | **reference** |
| --- | --- | --- | --- | --- | --- |
| ACTH* | mouse | clone 02A3 | DAKO | 1:2000 | [^9^](#_ENREF_9) |
| FSH | mouse | clone AB-3 | Thermo Fischer Scientific | 1:500 | [^9^](#_ENREF_9) |
| GH* | mouse | clone 54/9 2A2 | Biogenex | 1:100 | [^9^](#_ENREF_9) |
| Ki-67 | rabbit | clone SP6 | Thermo Fischer Scientific | 1:200 | [^9^](#_ENREF_9) |
| LH | mouse | clone Ab-1 | Thermo Fischer Scientific | 1:2000 | [^9^](#_ENREF_9) |
| SHH* | rabbit | ab73958 | Abcam | 1:100 |  |
| TSH | mouse | clone 0042 | DAKO | 1:500 | [^9^](#_ENREF_9) |
| PRL* | mouse | clone AB-2 | Thermo Fischer Scientific | 1:5000 | [^9^](#_ENREF_9) |

* For double immunofluorescent stainings secondary Cy2-labeled goat anti-mouse (1:100) and Cy3-labeled goat anti-rabbit antibodies (1:200) (Dianova) were used. Nuclei were counterstained with Hoechst 33342 at a concentration of 500 ng/ml for 5 minutes.

**Supplemental Figures**

**
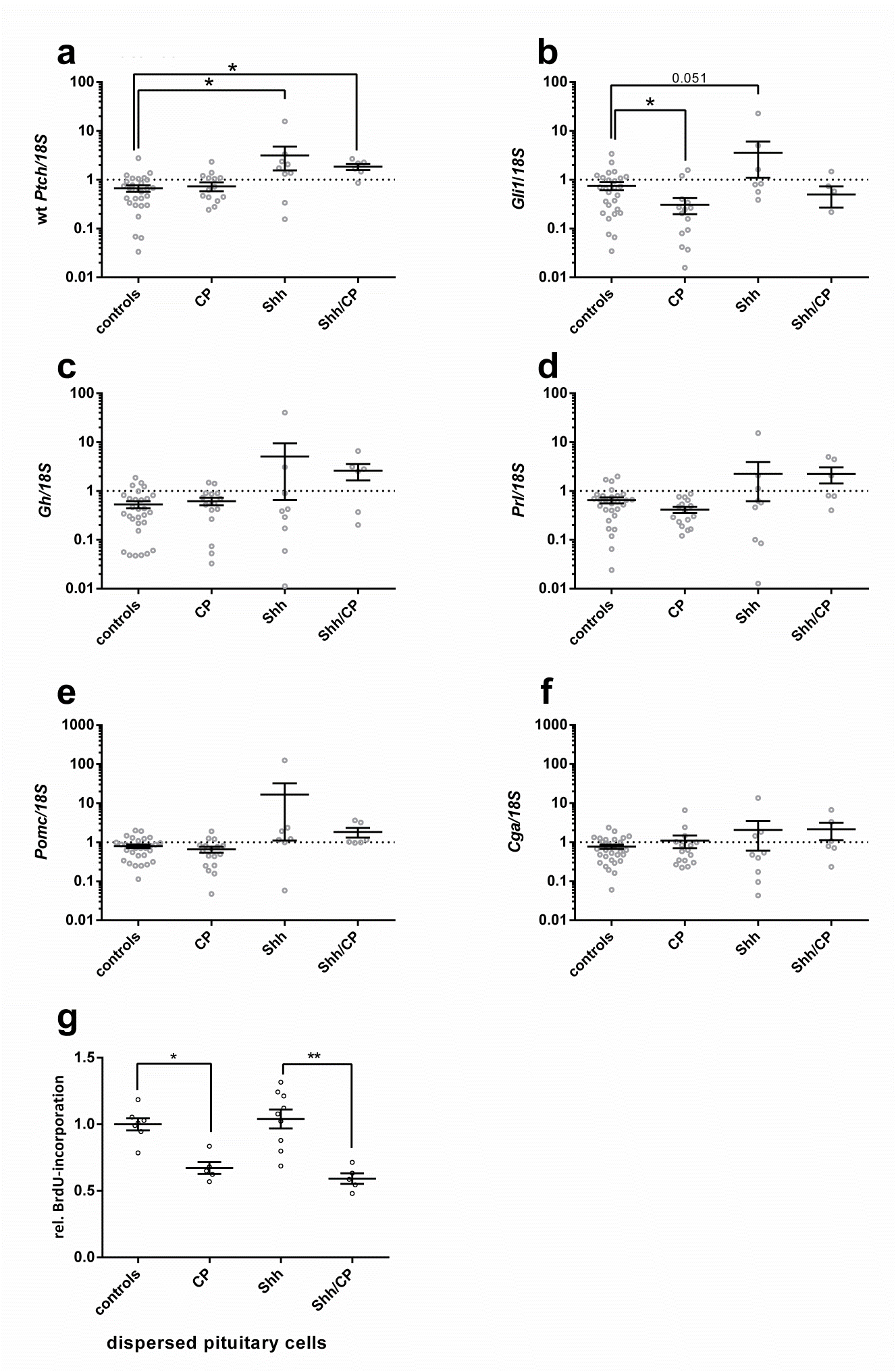
**

**Supplemental Figure S1: Effects of *ex vivo* modulation of Hh signaling in murine pituitaries on the expression of pituitary hormones and proliferation. (a-f)** Relative wt *Ptch*, *Gli1*, *Gh*, *Prl*, *Pomc* and *Glycoprotein hormones alpha chain* (*Cga*) expression levels and **(g)** BrdU incorporation assays from *ex vivo* cultured **(a-f)** *Ptch^flox/flox^* pituitary glands or **(g)** dispersed cells with and without cyclopamine (CP) and/or recombinant Shh-N (1 µg/ml) treatment (Shh). **(b-g)** Open circles indicate biological replicates measured in triplicates. Controls include vehicle-treated *Ptch^flox/flox^ CreERT2^+/-^*, tamoxifen- or vehicle-treated *Ptch^flox/flox^* and untreated pituitary glands/cells of both genotypes. **(b-f)** n_controls_=28 (14 females, 14 males), n_CP_=16 (7 females, 9 males), n_Shh_=9 (5 females, 4 males), n_Shh/CP_=6 (2 females, 4 males); Data shown in **(h)** represent 3 independent experiments. Expression levels and BrdU incorporation of the controls were set to 1. Horizontal lines, mean +/-SEM; * p<0.05, ** p<0.01.


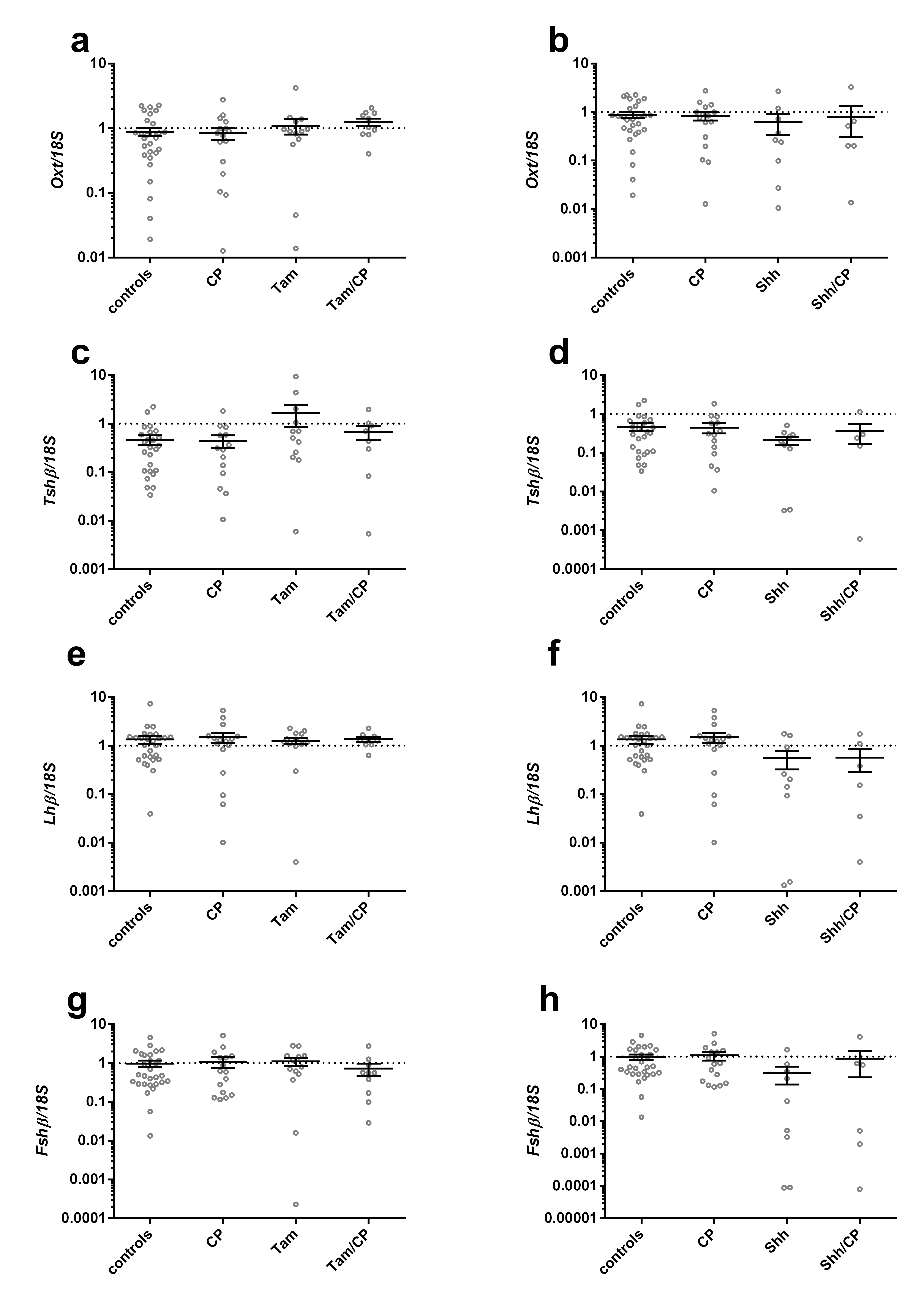


**Supplemental Figure S2: Effects of *ex vivo* modulation of Hh signaling in murine pituitaries on the expression of *Oxt*, *Tshβ*, *Lhβ* or *Fshβ*.** Relative *Oxt*, *Tshβ*, *Fshβ* and *Lhβ* expression levels of **(a, c, e, g)** *in vitro* recombined *Ptch^flox/flox^ CreERT2^+/-^* (Tam) and control pituitary glands with and without cyclopamine treatment (CP) and **(b, d, e, h)** of *in vitro* cultured *Ptch^flox/flox^* pituitary glands with and without cyclopamine (CP) and/or recombinant Shh-N (1 µg/ml) treatment (Shh). Open circles indicate biological replicates measured in triplicates. Controls include vehicle-treated *Ptch^flox/flox^ CreERT2^+/-^*, tamoxifen- or vehicle-treated *Ptch^flox/flox^* and untreated pituitary glands of both genotypes. Expression levels of the controls were set to 1. n_controls_=28 (14 females, 14 males), n_CP_=16 (7 females, 9 males), n_Tam_=13 (7 females, 6 males), n_Tam/CP_=10 (5 females, 5 males), n_Shh_=9 (5 females, 4 males), n_Shh/CP_=6 (2 females, 4 males); Horizontal lines, mean +/-SEM.

**
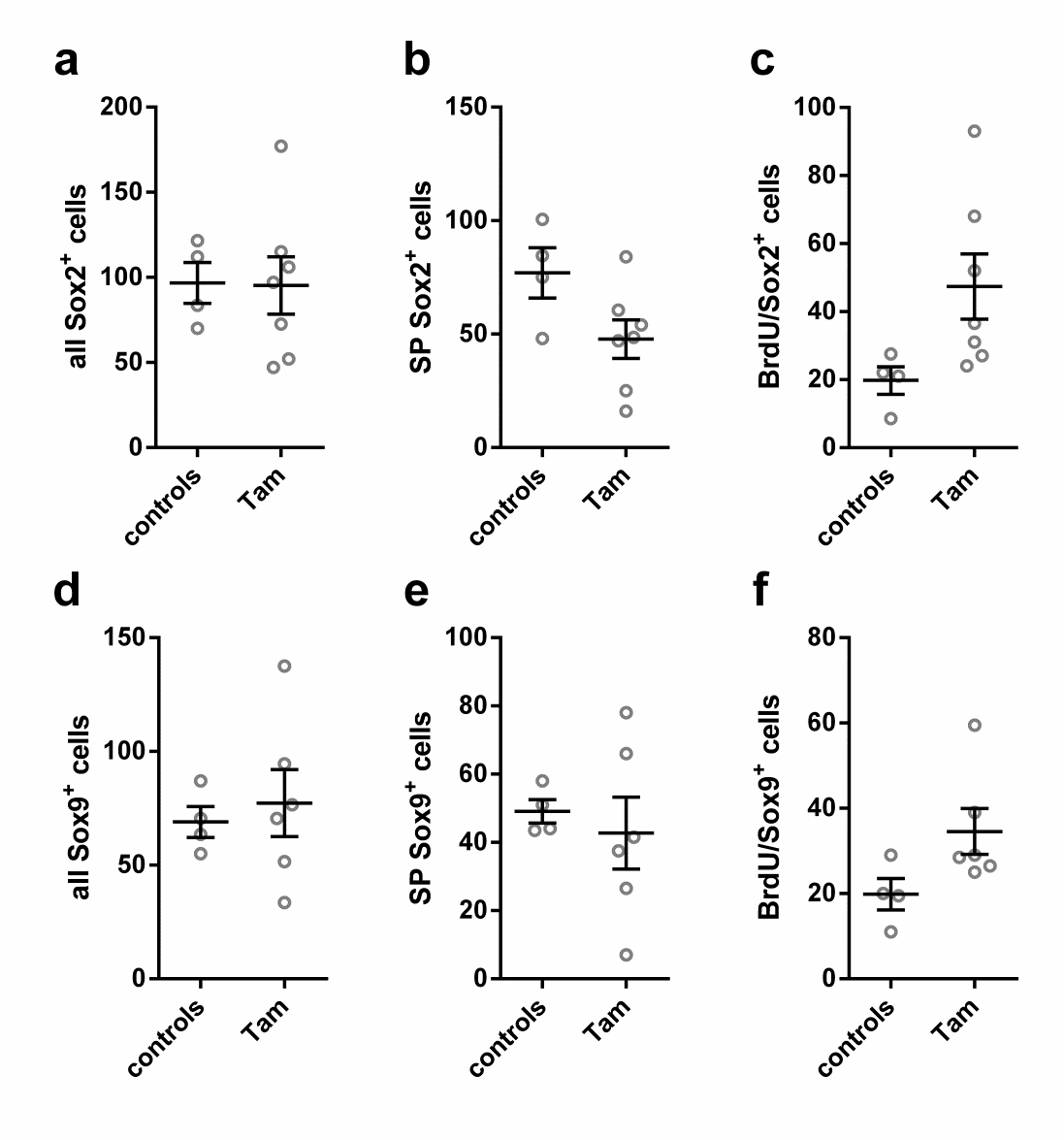
**

**Supplemental Figure S3: Absolute cell numbers of Sox2^+^ and Sox9^+^ cells of the anterior lobes of cultured murine pituitary explants.** Absolute cell numbers of **(a)** all Sox2^+^, **(b)** single-positive Sox2 (SP Sox2^+^), **(c)** double-positive BrdU/Sox2^+^ cells, **(d)** all Sox9^+^, **(e)** single-positive Sox9 (SP Sox9^+^), **(f)** double-positive BrdU/Sox9^+^ cells of the anterior lobe of immunofluorescent double stained tamoxifen-treated *Ptch^flox/flox^CreERT2^+/-^* (Tam) and control pituitary explants. Open circles indicate biological replicates counted in duplicates. As controls tamoxifen-treated *Ptch^flox/flox^* pituitary explants were analyzed. **(a-c)** n_controls_=4 (1 female, 3 males), n_Tam_=7 (6 females, 1 male), **(d-f)** n_controls_=4 (1 female, 3 males), n_Tam_=6 (5 females, 1 male). Horizontal lines, mean +/-SEM.


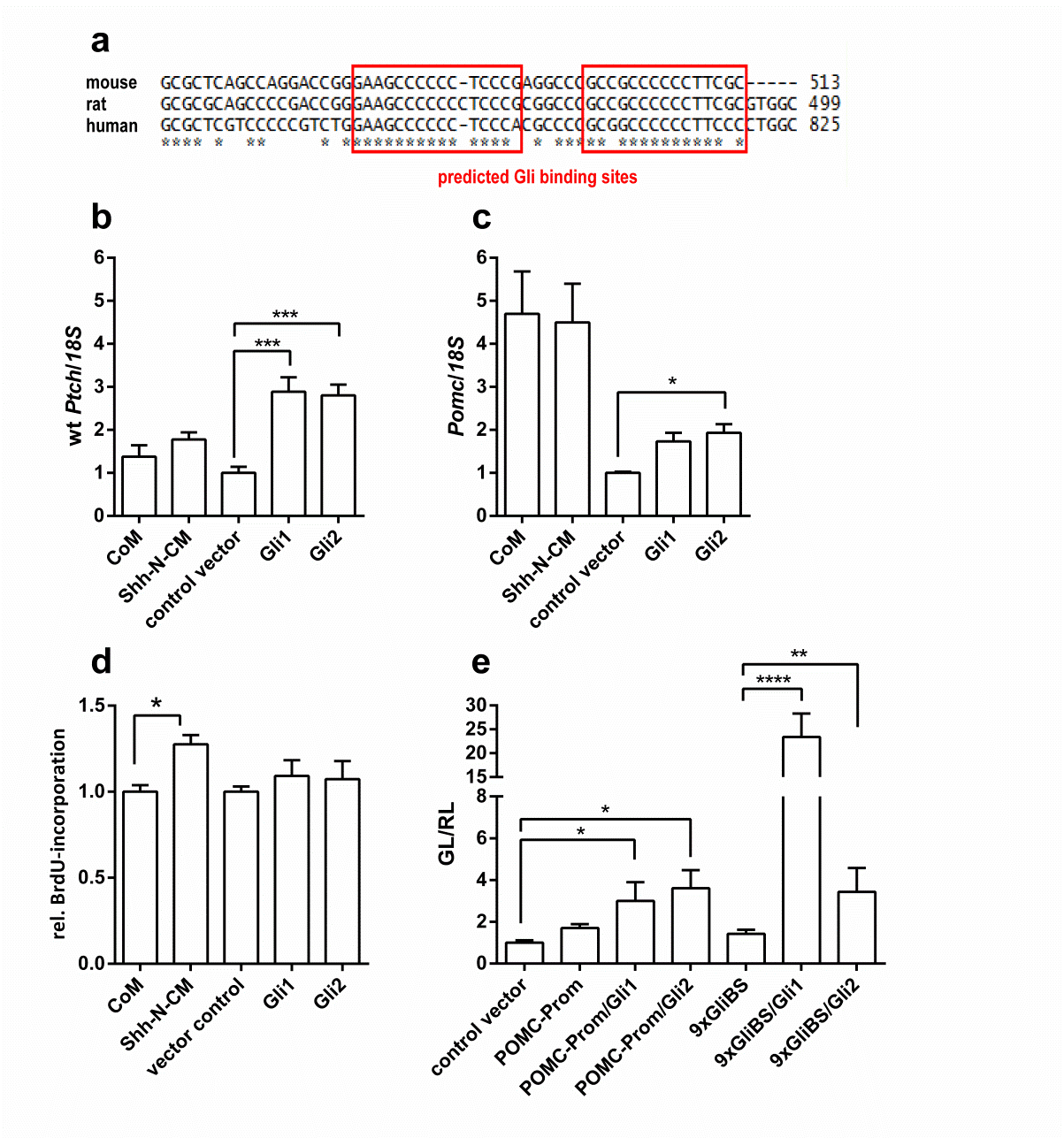


**Supplemental Figure S4: Gli-mediated activation of the murine and the human *Pomc/POMC* promoter. (a)** Sequence analyses using MatInspector ([http://www.genomatix.de](http://www.genomatix.de/)) revealed two conserved Gli binding sites upstream of the first ATG in the genomic sequences of the murine (GeneID: 18976; 3268-3283 bp; 3290-3305 bp), rat (GeneID: 24664; 3375-3390 bp; 3397-3413 bp) and human *Pomc/POMC* genes (GeneID: 5443; 3915-3930 bp; 3937-3952 bp). **(b and c)** Relative quantification of wt *Ptch* and *Pomc* expression levels and **(d)** BrdU incorporation in AtT-20 cells after Shh stimulation (Shh-N-CM) or transfection with either Gli1*-* or Gli2-expression plasmids cultured in F-12K medium. For preparation of Shh-N-CM or control media (CoM) see Material and Method section. **(e)** Dual luciferase-based analyses of the human *POMC* promoter activity after co-transfection of AtT-20 cells with a *POMC* promoter reporter plasmid (POMC-Prom) and either Gli1- or Gli2-expression vectors. Co-transfection of the 9xGliBS vector [^12^](#_ENREF_12) and Gli1- or Gli2-expression plasmids served as controls. Data represent at least 3 independent experiments measured in triplicates. Mean +/-SEM; * p<0.05, ** p<0.01, *** p<0.001, **** p<0.0001. For the experimental design and plasmids see Supplemental Material.


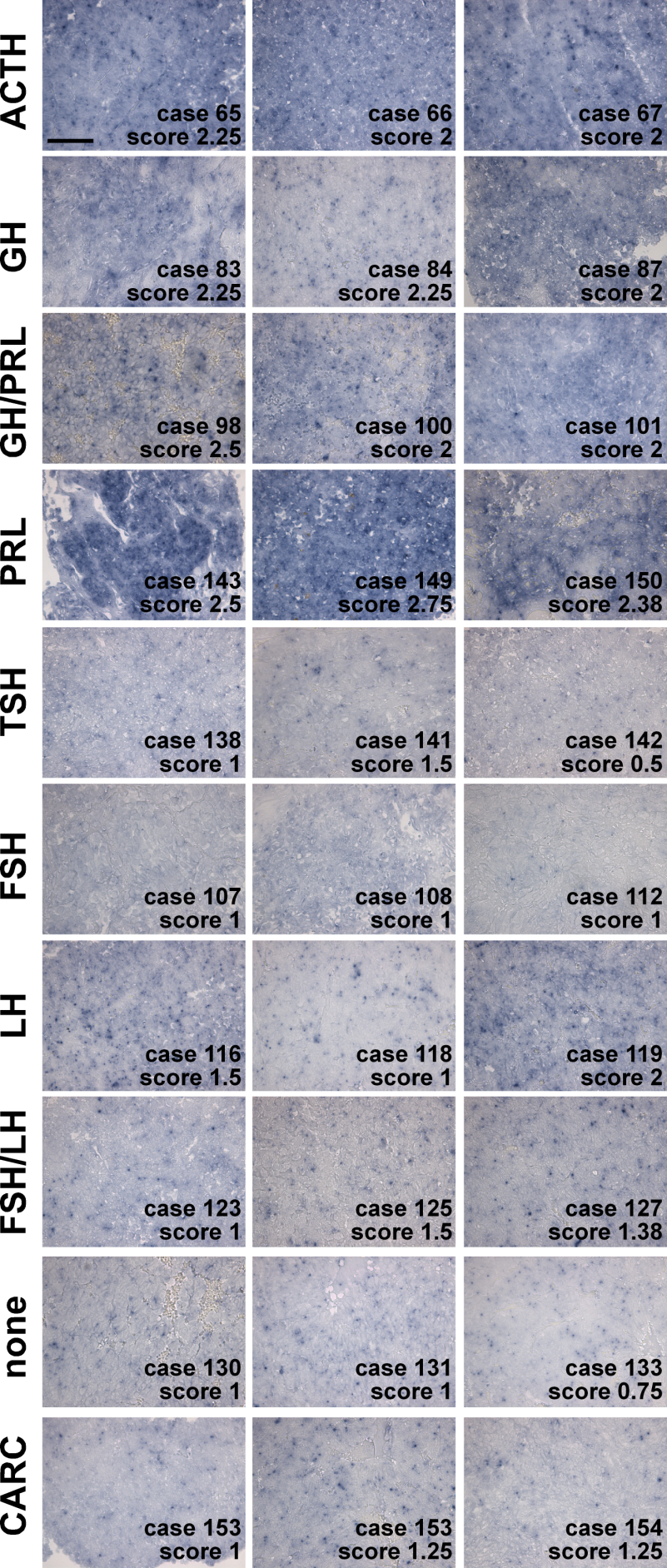


**Supplemental Figure S5: Representative *GLI1* *in situ* hybridizations of pituitary tumors.** ACTH positive, GH positive, mixed GH/PRL positive and PRL positive adenomas showed highest *GLI1* expression level whereas all other subtypes including pituitary carcinoma were moderately or low positive for *GLI1* expression. For case numbers see table 2. ACTH, adreno-corticotrophic hormone; GH, growth hormone; PRL, prolactin; TSH, thyroid stimulating hormone; FSH, follicle stimulating hormone; LH, luteinizing hormone; CARC, carcinoma. Tissue sections hybridized with *GLI1* sense riboprobes served as controls and were always negative (data not shown). Scale bar: 100 µm.


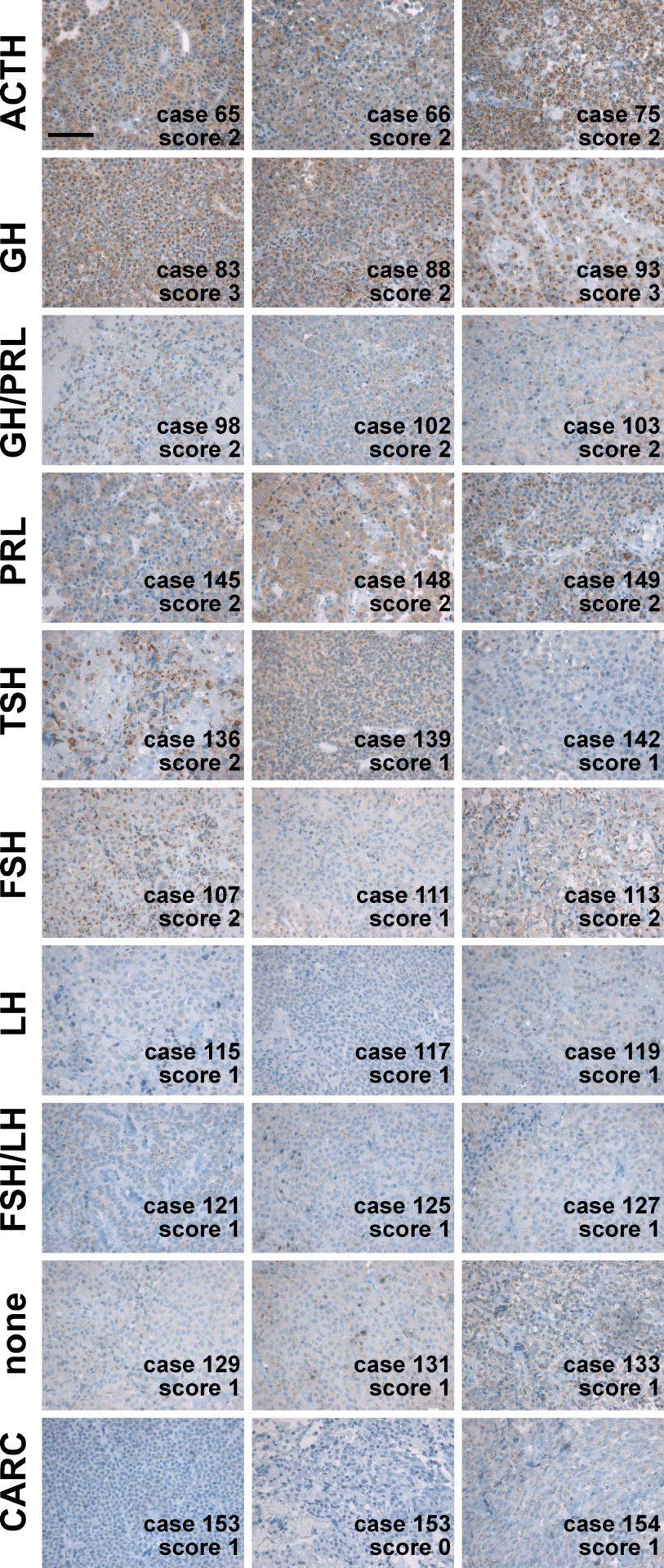


**Supplemental Figure S6: Representative immunohistological SHH stainings of pituitary tumors.** ACTH positive and GH positive adenomas showed highest SHH levels. Mixed GH/PRL positive, PRL positive and TSH positive adenomas were moderately positive for SHH. All other subtypes including pituitary carcinoma were low positive or negative for SHH. For case numbers see table 2. ACTH, adreno-corticotrophic hormone; GH, growth hormone; PRL, prolactin; TSH, thyroid stimulating hormone; FSH, follicle stimulating hormone; LH, luteinizing hormone; CARC, carcinoma. Scale bar: 100 µm.

**Supplemental References**

1 Man, P. S., Wells, T. & Carter, D. A. Cellular distribution of Egr1 transcription in the male rat pituitary gland. *J Mol Endocrinol* **53**, 271-280, doi:10.1530/JME-14-0158 (2014).

2 Nitzki, F. *et al.* Tumor stroma-derived Wnt5a induces differentiation of basal cell carcinoma of Ptch mutant mice via CaMKII. *Cancer Res* **70**, 2739-2748 (2010).

3 Gong, X., Wang, Y., Zeng, J., Li, S. & Luo, Y. Computational identification and experimental validation of microRNAs binding to the fragile X syndrome gene Fmr1. *Neurochem Res* **40**, 109-117, doi:10.1007/s11064-014-1471-3 (2015).

4 Andoniadou, C. L. *et al.* Sox2(+) stem/progenitor cells in the adult mouse pituitary support organ homeostasis and have tumor-inducing potential. *Cell Stem Cell* **13**, 433-445, doi:10.1016/j.stem.2013.07.004 (2013).

5 Niemann, C. *et al.* Indian hedgehog and beta-catenin signaling: role in the sebaceous lineage of normal and neoplastic mammalian epidermis. *Proc Natl Acad Sci U S A* **100 Suppl 1**, 11873-11880, doi:10.1073/pnas.1834202100 (2003).

6 Kaushik, S. *et al.* Loss of autophagy in hypothalamic POMC neurons impairs lipolysis. *EMBO Rep* **13**, 258-265, doi:10.1038/embor.2011.260 (2012).

7 Tsakiridis, A. *et al.* Distinct Wnt-driven primitive streak-like populations reflect in vivo lineage precursors. *Development* **141**, 1209-1221, doi:10.1242/dev.101014 (2014).

8 Yamamizu, K., Schlessinger, D. & Ko, M. S. SOX9 accelerates ESC differentiation to three germ layer lineages by repressing SOX2 expression through P21 (WAF1/CIP1). *Development* **141**, 4254-4266, doi:10.1242/dev.115436 (2014).

9 Buslei, R. *et al.* Activation and regulation of endogenous retroviral genes in the human pituitary gland and related endocrine tumors. *Neuropathol Appl Neurobiol*, doi:10.1111/nan.12136 (2014).

10 Stasikowska-Kanicka, O., Wagrowska-Danilewicz, M., Bialek, I. & Danilewicz, M. The immunoexpression of Shh, Smo and Gli2 in Helicobacter pylori positive and negative gastric biopsies. *Pol J Pathol* **63**, 25-30 (2012).

11 Witt, R. M. *et al.* Heparan sulfate proteoglycans containing a glypican 5 core and 2-O-sulfo-iduronic acid function as Sonic Hedgehog co-receptors to promote proliferation. *J Biol Chem* **288**, 26275-26288, doi:10.1074/jbc.M112.438937 (2013).

12 Beer, C., Buhr, P., Hahn, H., Laubner, D. & Wirth, M. Gene expression analysis of murine cells producing amphotropic mouse leukaemia virus at a cultivation temperature of 32 and 37 degrees C. *J Gen Virol* **84**, 1677-1686 (2003).
